# Lyssavirus matrix protein stabilizes RGS4 to suppress ferroptosis via incomplete autophagy and iron metabolic reprogramming

**DOI:** 10.1371/journal.ppat.1014726

**Published:** 2026-10-21

**Authors:** Jianqing Zhao, Pingping Chen, Meixin Sun, Chuan Liang, Zhen F. Fu, Ling Zhao, Ming Zhou

**Affiliations:** 1 National Key Laboratory of Agricultural Microbiology, Huazhong Agricultural University, Wuhan, China; 2 Key Laboratory of Preventive Veterinary Medicine of Hubei Province, College of Veterinary Medicine, Huazhong Agricultural University, Wuhan, China; 3 Frontiers Science Center for Animal Breeding and Sustainable Production, Wuhan, China; 4 Hubei Hongshan Laboratory, Wuhan, China; Seoul National University School of Biological Sciences, KOREA, REPUBLIC OF

## Abstract

Rabies virus (RABV), the prototypical lyssavirus, suppresses neuronal ferroptosis through lipid droplet (LD) biogenesis, which is a critical immune evasion strategy we previously identified. However, the mechanistic link between LD formation and ferroptosis regulation during RABV infection remains poorly defined. Here, we report that RABV upregulate Regulator of G Protein Signaling 4 (RGS4), a ferroptosis-associated regulator, and this induction is strictly dependent on triglyceride synthesis and subsequent LD formation. Functionally, genetic or pharmacological inhibition of RGS4 elevates ferroptosis susceptibility, thereby restricting viral replication. Mechanistically, RGS4 depletion reduces HERC2, impairing the assembly of branched K48/K63 ubiquitin chains on NCOA4, which is essential for its proteasomal degradation, thereby enhancing NCOA4 stability. Stabilized NCOA4 promotes ferritinophagy, leading to degradation of ferritin heavy chain (FTH) and ultimately inducing ferroptosis. Critically, we identified that the lyssavirus matrix (M) protein directly counteracts this pathway: M protein inhibits RGS4 degradation via the Cys/N-degron system through incomplete autophagy, stabilizing RGS4 to maintain HERC2 expression, promote NCOA4 ubiquitination, and attenuate ferritinophagy. This mechanism reduces ferroptosis susceptibility and enables viral replication. Our findings unveil a novel lyssavirus-specific regulatory axis, RGS4-HERC2-NCOA4-FTH, that modulates ferroptosis, highlighting the critical role of ferroptosis in lyssavirus infection and pathogenesis.

## Author summary

Rabies virus (RABV) evades the immune system by suppressing neuronal ferroptosis through lipid droplet (LD) biogenesis. Here, we uncover a novel regulatory axis through which RABV orchestrates this process: RABV infection upregulate the ferroptosis-associated regulator RGS4, which depends on triglyceride synthesis and LD formation. Mechanistically, RGS4 maintains the expression of the E3 ubiquitin ligase HERC2, which promotes branched K48/K63-linked ubiquitination and subsequent proteasomal degradation of NCOA4. This inhibits ferritinophagy and blocks ferritin heavy chain (FTH) degradation, thereby reducing ferroptosis susceptibility and enabling viral replication. Critically, RABV matrix (M) protein directly stabilizes RGS4 by interfering with its degradation via the Cys/N-degron system through incomplete autophagy. Furthermore, we identified this mechanism as a conserved pathway across lyssaviruses. In summary, our findings demonstrate that lyssavirus hijacks the RGS4-HERC2-NCOA4-FTH axis to reduce ferroptosis susceptibility, thereby revealing how lyssavirus M protein modulates ferroptosis to facilitate viral replication. This work elucidates a previously undefined mechanism of viral evasion and provides novel avenues for developing interventions against lyssavirus.

## Introduction

The *Lyssavirus* genus, belonging to the *Rhabdoviridae* family, represents the causative agent of neurotropic zoonotic infectious diseases [1]. The lyssavirus genome consists of a single negative-sense RNA strand, which encodes five proteins in the following order: nucleoprotein (N), phosphoprotein (P), matrix protein (M), glycoprotein (G), and large RNA polymerase protein (L) [2,3]. Currently, the lyssavirus genus encompasses several species, including Rabies virus (RABV), Australian bat lyssavirus (ABLV), Duvenhage lyssavirus (DUVV), European bat lyssavirus (EBLV), Lagos bat lyssavirus (LBV), and Mokola lyssavirus (MOKV) [4]. Among these, RABV has been studied extensively. During the budding process, the RABV M protein effectively facilitates viral budding via autophagosomes and lipid droplets, thereby further promoting viral replication [5]. This highlights the pivotal role of host lipid metabolism in viral replication. Furthermore, virus-induced metabolic reprogramming of host cells further modulates the intracellular immune response, ultimately altering cell fate and promoting viral replication [6].

The synthesis of neutral lipids in cells primarily occurs in the endoplasmic reticulum, and the produced neutral lipids are subsequently trafficked to lipid droplets (LDs) for storage [7,8]. Triglycerides (TG) and cholesteryl esters are the two major components of LDs, with TG being more abundant in the central nervous system (CNS) [7,9,10]. Diacylglycerol acyltransferases (DGAT1 and DGAT2) are rate-limiting enzymes that govern TG synthesis [11–13]. Inhibiting their activity can effectively reduce neuronal LD accumulation. The brain is not only a lipid-rich region in the body but also exhibits significantly higher iron levels and oxygen metabolism compared to other organs [14]. Consequently, restricting LD formation facilitates iron-catalyzed peroxidation of polyunsaturated fatty acids (PUFAs) in membrane phospholipids through enzymatic and non-enzymatic reactions, thereby increasing ferroptosis susceptibility [15,16]. During RABV infection, LD formation effectively sequesters free fatty acids, thereby limiting PUFA peroxidation and reducing ferroptosis susceptibility, which in turn promotes viral replication [17].

Ferroptosis is an iron-dependent form of programmed cell death [18]. Cellular iron uptake occurs via various routes, including the transferrin receptor (TFR) pathway, contributing to the formation of a labile iron pool in the cytoplasm [19]. A portion of this iron is sequestered within ferritin, while the remainder participates in essential metabolic processes [20]. The ferritin heavy chain (FTH) undergoes degradation through NCOA4-mediated ferritinophagy, releasing iron ions and thereby elevating intracellular iron levels [21,22]. This elevation promotes lipid peroxidation and enhances cellular ferroptosis susceptibility.

Regulator of G-protein signaling (RGS) proteins are a class of regulators that govern G-protein coupled receptor (GPCR) signaling through their “braking” function [23,24]. These proteins are currently classified into four subfamilies (A/RZ, B/R4, C/R7, D/R12) based on domain architecture and sequence conservation [25]. RGS4, a member of the B/R4 subfamily, is characterized by an α-helix and an “RGS box” domain (also known as the RH domain) [26,27]. Downregulation of RGS4 expression serves as a regulator for ferroptosis [28,29]. Mechanistically, RGS4 upregulates SLC7A11 to enhance glutamate transport and activate N-methyl-D-aspartate receptor (NMDAR) signaling [30]. Furthermore, RGS4 directly suppresses PERK/ERK phosphorylation and inhibits the AKT-mTOR axis [31,32]. However, the mechanisms underlying RGS4 regulation during ferroptosis and its precise role in mediating ferroptotic induction remain unresolved.

In this study, we found that lyssavirus infection effectively promotes LD formation and upregulates RGS4. Inhibiting LD formation leads to a significant decrease in RGS4 levels and an increase in ferroptosis susceptibility. RNA-Seq analysis revealed that the loss of RGS4 markedly disrupted cellular iron metabolism by promoting NCOA4-dependent ferritinophagy, which significantly enhanced the FTH degradation and thereby increased ferroptosis susceptibility. Furthermore, we demonstrated that lyssavirus M stabilizes RGS4 expression by the Cys/N-degron pathway through incomplete autophagy. Upon lyssavirus infection, this mechanism suppresses ferroptosis via RGS4 stabilization, thereby promoting viral replication. Our work collectively demonstrates a novel mechanism by which lyssavirus M modulates PTMs to govern ferroptosis and enhance viral replication, providing a mechanistic foundation for exploring host-virus regulatory interactions.

## Results

### DGAT-mediated lipid droplets sustain RGS4 expression in RABV-infected neurons

To explore the molecular link between LD biogenesis and ferroptosis during lyssavirus infection, an RNA-seq assay was used to analyze gene expression after inhibition of DGAT activity during RABV (a typical lyssavirus) infection. Among the top 50 differentially expressed genes, RGS4, a known ferroptosis-associated regulator, exhibited the most significant downregulation (Fig 1A). Furthermore, validations across two neuronal models including primary neurons and mouse-derived Neuro-2a (N2a) cells were carried out, and the results demonstrated that RGS4 mRNA levels were consistently reduced following treatment with a DGAT inhibitor in the context of RABV infection (Fig 1B-C). Consistently, RGS4 protein levels were diminished in infected primary neurons (Fig 1D-E) and N2a cells (Fig 1F-G) upon DGAT inhibition. Collectively, these data establish that LD formation is required for maintaining RGS4 expression during RABV infection in neuronal cells.

**Fig 1:**
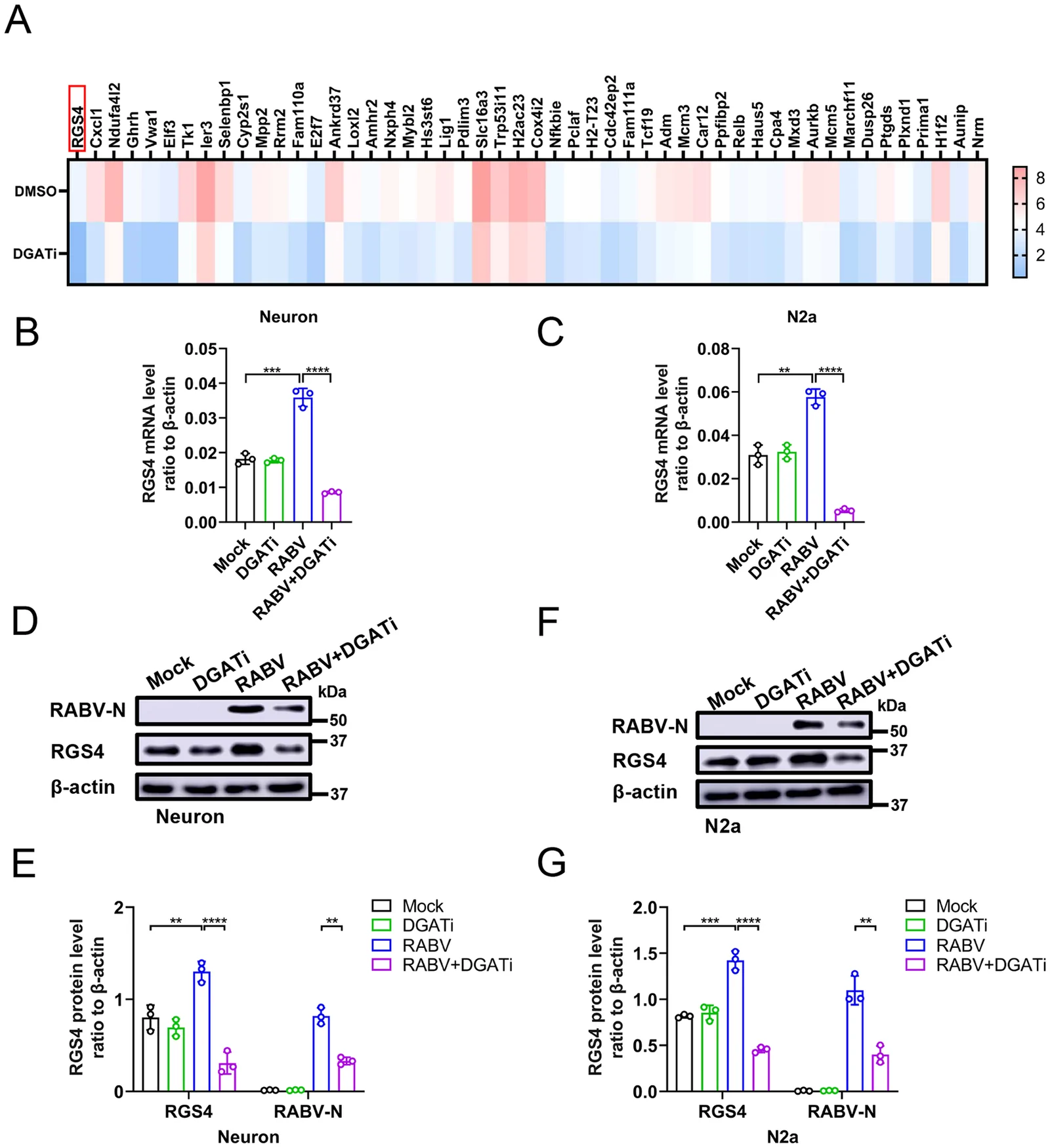
Targeting DGAT to inhibit RABV infection significantly decreases RGS4 expression. **(A)** RNA-seq heatmap showing significantly down-regulated genes in RABV-infected (MOI = 0.1, 48 **h)** N2a cells with DGAT inhibitors (A922500, 8 μg/mL; PF06424439, 10 μg/mL). Quantification of RGS4 mRNA levels in primary neurons (B) and N2a cells (C) infected with RABV ± DGAT inhibitors. **(D)** Immunoblot analysis and (E) quantification of RGS4 and RABV-N protein levels in primary neurons (MOI = 0.1, 48 **h)**. **(F)** Immunoblot analysis and (G) quantification of RGS4 and RABV-N protein levels in N2a cells (MOI = 0.1, 48 **h)**. Error bars represent mean ± SD (n = 3). *P < 0.05, **P < 0.01, ***P < 0.001, ****P < 0.0001 (unpaired two-tailed Student’s t-test).

### RGS4 depletion restricts RABV production by impairing viral replication

To define the functional role of RGS4 in RABV replication, we generated RGS4-knockout (RGS4 KO) N2a cells via CRISPR-Cas9-mediated genome editing. RGS4 ablation resulted in a significant reduction in viral titers at both 24 and 48 hours post-infection (hpi), compared to wild-type (WT) controls (Fig 2A). This antiviral phenotype was confirmed by decreased RABV nucleoprotein (N) expression at both the protein (Fig 2B-C) and genomic RNA (gRNA) levels (Fig 2D). Consistent with genetic ablation, pharmacological inhibition of RGS4 using the small-molecule inhibitor CCG-63802 dose-dependently suppressed RABV production across multiple multiplicities of infection (MOIs) (S1A-B Fig).

**Fig 2:**
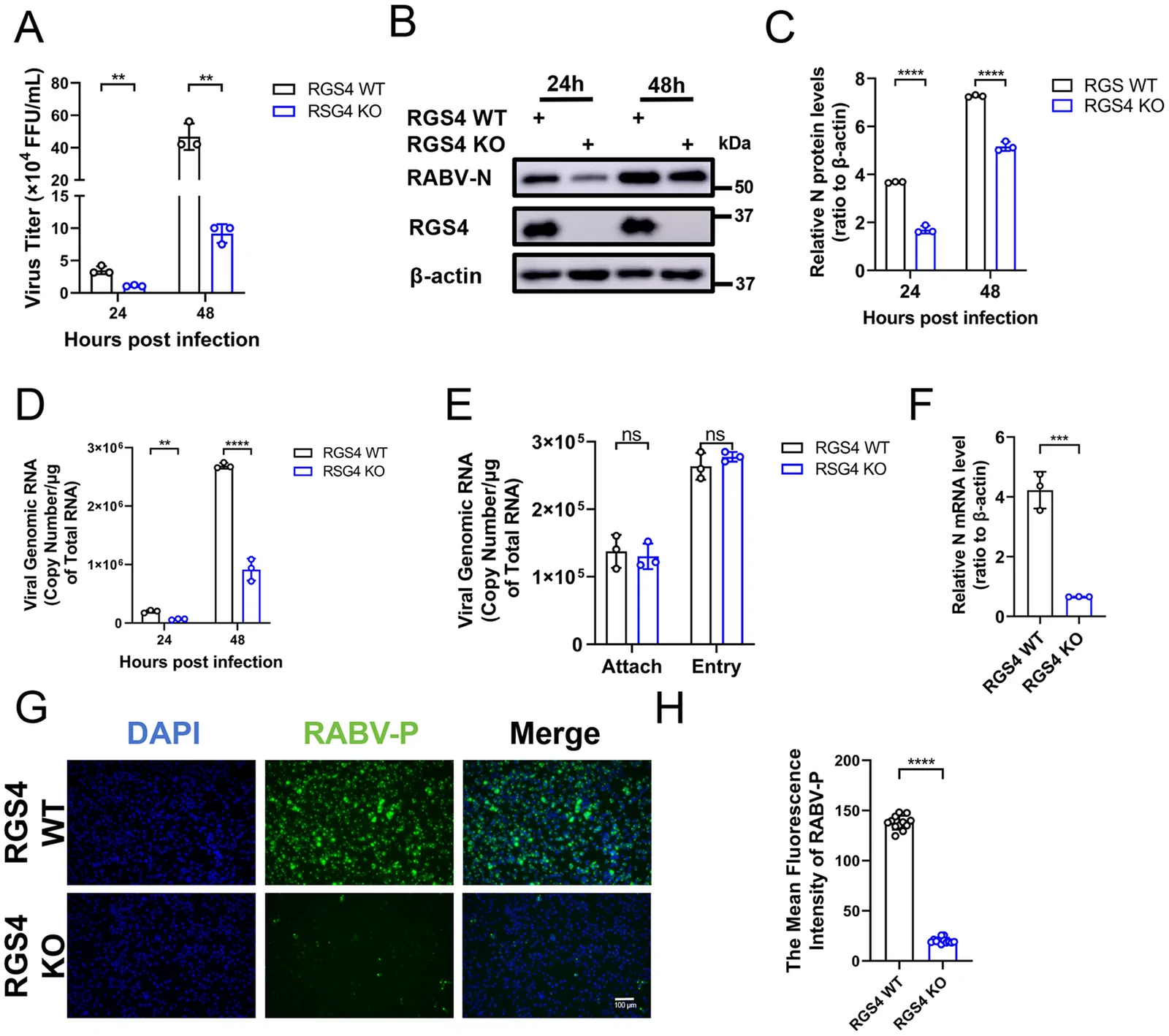
RGS4 reduction restricts RABV replication. **(A)** Viral titers in RGS4 WT and RGS4 KO N2a cells infected with RABV (MOI = 0.1, 24 h and 48 **h)**. **(B)** Immunoblot analysis and (C) quantification of RABV-N protein levels in RGS4 WT and RGS4 KO N2a cells infected with RABV. **(D)** Quantification of viral genome RNA in RGS4 WT and RGS4 KO N2a cells infected with RABV. **(E)** RABV genomic RNA levels in RGS4 WT and RGS4 KO N2a cells infected with RABV (attachment/entry phase: 4 °C for 1 h or 37 °C for 2 h, MOI = 0.1). **(F)** RABV-N mRNA levels in RGS4 WT and RGS4 KO N2a cells infected with RABV (replication phase: 24 h post-infection, MOI = 0.1). **(G)** Immunofluorescence analysis and (H) quantification of RABV-P protein (green) in RGS4 WT and RGS4 KO N2a cells infected with RABV (G, scale bar: 100 μm). Error bars represent mean±SD (n = 3). **P < 0.01, ***P < 0.001, ****P < 0.0001 (unpaired two-tailed Student’s t-test).

To map the stage of the viral life cycle targeted by RGS4, we systematically evaluated viral adsorption, internalization, and replication. Viral replication was markedly impaired in RGS4 KO cells without affecting attachment or entry (Fig 2E-F), as further confirmed by reduced fluorescence intensity of RABV P-expressing foci (Fig 2G-H). Collectively, these data establish that RGS4 is an essential host factor for efficient RABV replication, with its depletion restricting viral production primarily by impairing the replication stage of the viral life cycle.

### RGS4 depletion restricts rabies virus replication by sensitizing neurons to ferroptosis

Given that reduced RGS4 expression has been previously linked to enhanced ferroptosis susceptibility [28], we hypothesized that RGS4 depletion restricts RABV replication by promoting ferroptosis in infected neuronal cells. To test this, we first measured cell viability and redox homeostasis in RGS4 KO cells following RABV infection. RGS4 KO cells exhibited significantly increased cell death and a reduced glutathione (GSH)/oxidized glutathione (GSSG) ratio compared to WT controls (Fig 3A-B), indicative of impaired antioxidant capacity. Moreover, consistent with ferroptosis induction, RGS4 KO cells showed marked elevations in cellular lipid peroxidation, labile ferrous iron (Fe^2+^) levels, and malondialdehyde (MDA) content upon RABV infection (Fig 3C-E). To confirm that these phenotypes were specifically due to RGS4 depletion, we performed a rescue experiment by re-expressing RGS4 in KO cells. RGS4 re-expression completely reversed the ferroptotic phenotype, restoring cell viability, reducing lipid peroxidation, and normalizing Fe^2+^ levels (Fig 3F-H). Furthermore, to determine whether ferroptosis is the predominant mode of cell death induced by RGS4 deletion, cells were treated with the ferroptosis inhibitor Ferrostatin-1, the apoptosis inhibitor Z-VAD-FMK, or the necroptosis inhibitor GSK872, as previously described [33]. We observed that treatment with Ferrostatin-1, rather than Z-VAD-FMK or GSK872, significantly suppressed the elevated cell death rate in RGS4 KO cells (Fig 3I). Meanwhile, the viral titer, viral genome copy number, and RABV-N protein expression levels were all rescued (Fig 3J-M). This further demonstrates that RGS4 deletion specifically restricts RABV replication by promoting ferroptosis. Moreover, treatment with Ferrostatin-1 attenuated the elevated cell death in RGS4-knockdown BSR cells (S2A Fig) and rescued both the viral titer and RABV-N protein expression levels (S2B-D Fig). Collectively, these data demonstrate that RGS4 reduction sensitizes neuronal cells to ferroptosis, thereby restricting RABV replication.

**Fig 3:**
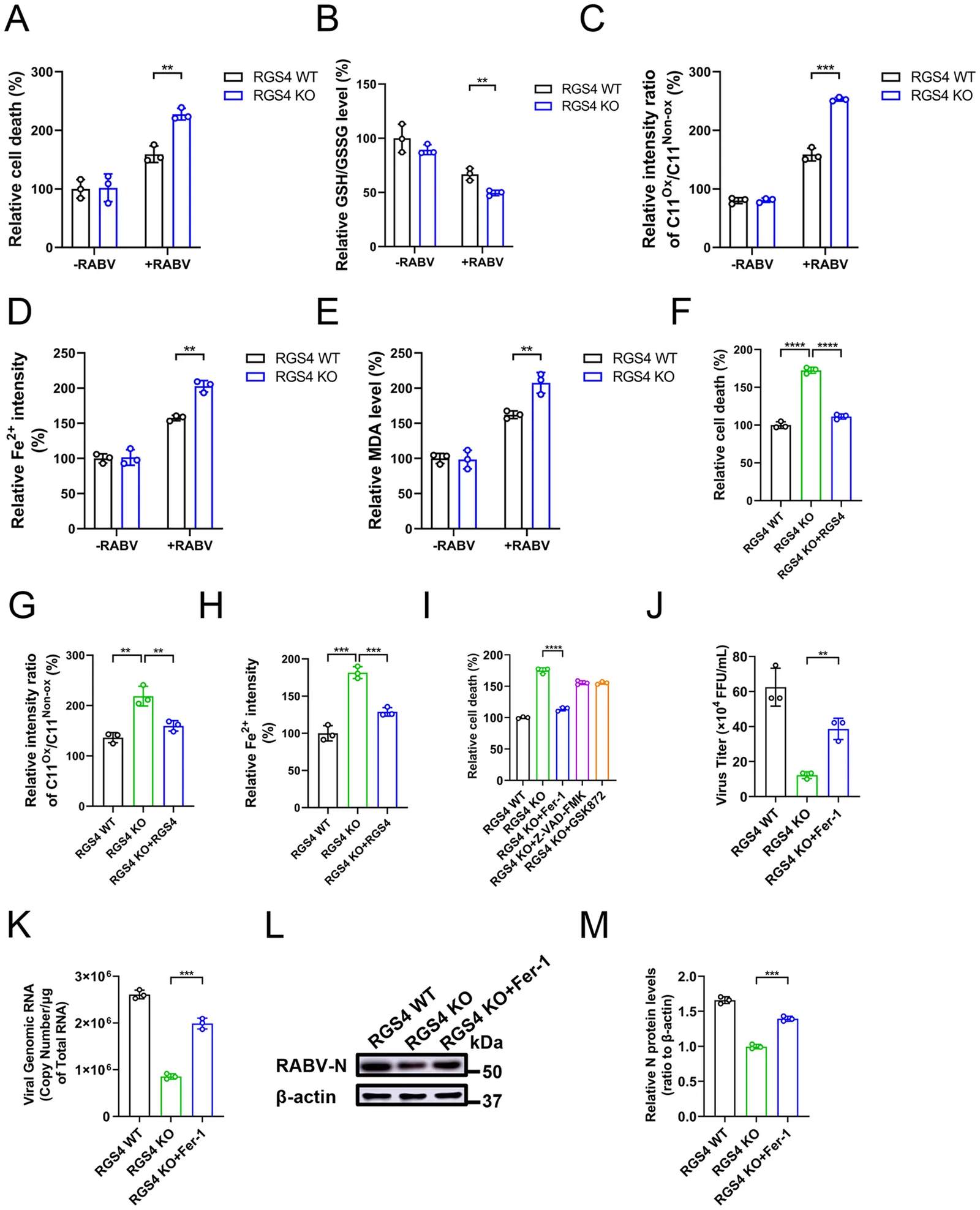
RGS4 reduction increases ferroptosis susceptibility in RABV infection. Quantification of cell death **(A)**, GSH/GSSG **(B)**, lipid peroxidation **(C)**, Fe^2+^ (D) and MDA (E) in RGS4 WT and RGS4 KO N2a cells infected with RABV (MOI = 0.1, 48 **h)**. Quantification of cell death **(F)**, lipid peroxidation (G) and Fe^2+^ (H) in indicated N2a cells infected with RABV (MOI = 0.1, 48 **h)**. **(I)** Quantification of cell death in RGS4 WT and RGS4 KO N2a cells treated with Ferrostatin-1 (10 μM), Z-VAD-FMK (25 μM) and GSK872 (2.5 μM) and infected with RABV. **(J)** Viral titers in RGS4 WT and RGS4 KO N2a cells treated with Ferrostatin-1 (10 μM) and infected with RABV. **(K)** Quantification of viral genome RNA in RGS4 WT and RGS4 KO N2a cells treated with Ferrostatin-1 and infected with RABV. **(L)** Immunoblot analysis and (M) quantification of RABV-N protein levels in RGS4 WT and RGS4 KO N2a cells treated with Ferrostatin-1 and infected with RABV. Error bars represent mean±SD (n = 3). **P < 0.01, ***P < 0.001, ****P < 0.0001 (unpaired two-tailed Student’s t-test or one-way ANOVA).

### RGS4 depletion promotes ferroptosis via NCOA4-mediated ferritinophagy

To delineate the molecular mechanism by which RGS4 depletion sensitizes cells to ferroptosis and restricts RABV replication, we performed RNA-seq on RABV-infected RGS4-WT and RGS4-KO N2a cells. Pathway enrichment analysis revealed significant dysregulation of ferroptosis-related genes, with 7 upregulated (e.g., CP, STEAP3, SAT2, LPCAT3, TFRC, GSS, NCOA4) and 7 downregulated genes (e.g., VDAC3, HMOX1, ACSL1, GCLC, SLC3A2, SLC7A11, ACSL6) (Fig 4A). Notably, over 50% of upregulated genes (CP, STEAP3, TFRC and NCOA4) were linked to iron metabolism, prompting us to hypothesize that RGS4 modulates RABV replication through regulation of cellular iron homeostasis. Focusing on iron metabolism, we confirmed that RGS4 KO cells exhibited significantly elevated NCOA4 mRNA levels (Fig 4B), a signature associated with enhanced ferritinophagy. At the protein level, RABV-infected RGS4 KO cells showed increased NCOA4 expression and an elevated LC3-II/LC3-I ratio, alongside a reduced abundance of the downstream NCOA4 target, FTH (Fig 4C-D). Immunofluorescence staining further validated accelerated ferritinophagy, as evidenced by enhanced colocalization of FTH with the lysosomal marker LAMP1 (Fig 4E-I). To establish functional causality, we performed shRNA-mediated knockdown of NCOA4 in RGS4 KO cells. This abrogated the RGS4 depletion-induced cell death and labile Fe^2+^ accumulation (Fig 4J-K), confirming NCOA4 as a key mediator. Consistent with this, treatment with the autophagy inhibitor chloroquine (CQ) rescued FTH degradation in RGS4-deficient cells (Fig 4L-M). Collectively, these data demonstrate that RGS4 reduction sensitizes neuronal cells to ferroptosis by promoting NCOA4-dependent ferritinophagy and subsequent disruption of iron homeostasis.

**Fig 4:**
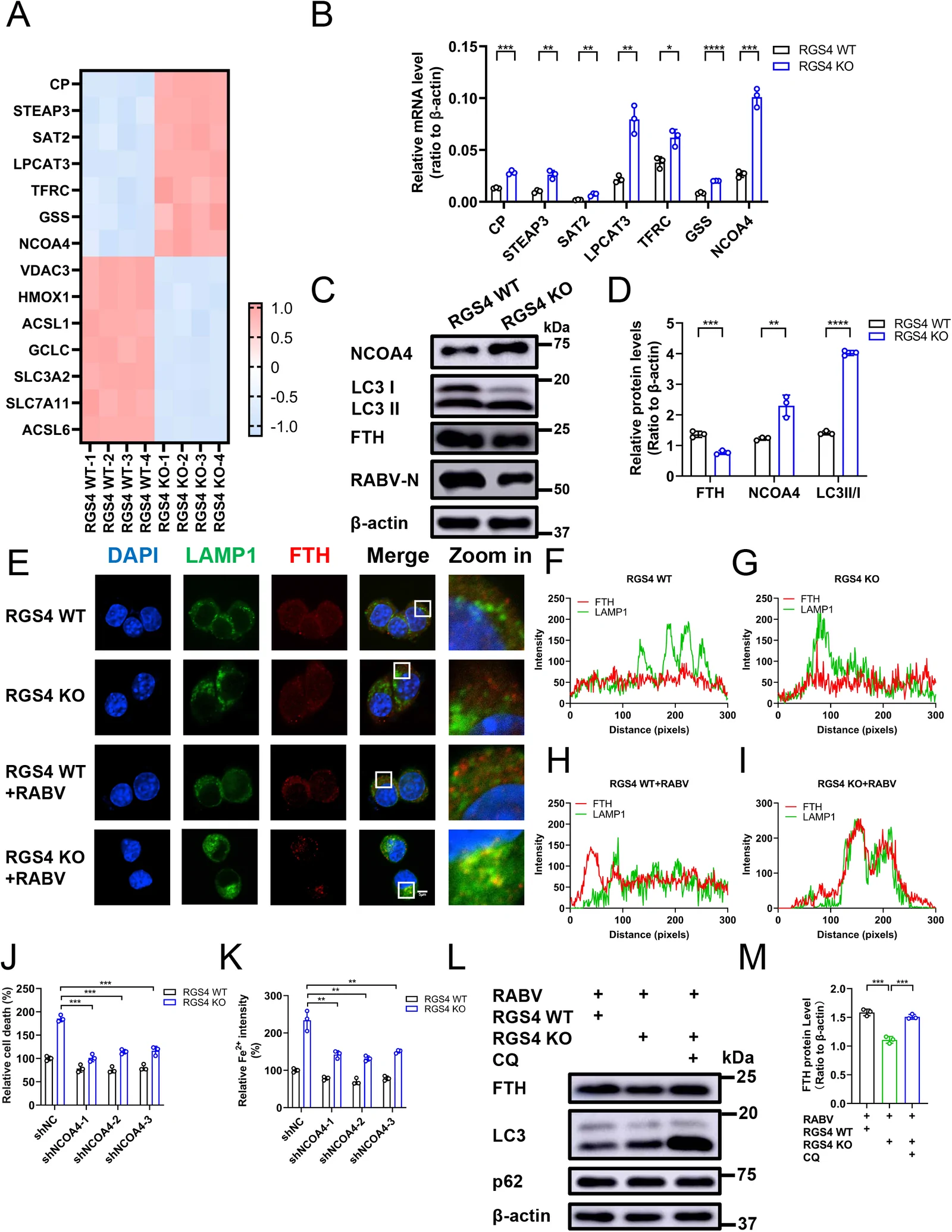
RGS4 reduction promotes NCOA4-mediated ferritinophagy to regulate ferroptosis susceptibility. **(A)** RNA-seq heatmap showing differentially expressed genes (DEGs) in iron metabolism in RGS4 WT and RGS4 KO N2a cells infected with RABV (MOI = 0.1, 48 **h)**. **(B)** Quantification of CP, STEAP3, SAT2, LPCAT3, TFRC, GSS and NCOA4 mRNA level in RGS4 WT and RGS4 KO N2a cells infected with RABV. **(C)** Immunoblot analysis and (D) quantification of NCOA4, LC3II/I and FTH protein level in RGS4 WT and RGS4 KO N2a cells infected with RABV. Representative images (E) and co-localization analysis (F-I) of FTH (red) and LAMP1 (green) co-localization (scale bar: 5 μm) in RGS4 WT and RGS4 KO N2a cells infected with RABV. Quantification of cell death (J) and Fe^2+^ (K) in RGS4 WT and RGS4 KO N2a cells infected with RABV and transfected with shNCOA4. **(L)** Immunoblot analysis and (M) quantification of FTH protein level in RGS4 WT and RGS4 KO N2a cells infected with RABV and treated with chloroquine (CQ, 10 μM). Error bars represent mean±SD (n = 3). *P < 0.05, **P < 0.01, ***P < 0.001 (unpaired two-tailed Student’s t-test or one-way ANOVA).

### RABV-M protein stabilizes RGS4 by inhibiting Cys/N-degron pathway-mediated autophagic degradation

To identify which RABV structural protein regulates RGS4 expression, we individually transfected N2a cells with plasmids encoding RABV nucleoprotein (N), phosphoprotein (P), matrix (M), or glycoprotein (G). Although N, P, M, and G proteins all exerted a stabilizing effect on RGS4, the M protein played the predominant role, as evidenced by its significantly greater potency compared with the other viral proteins (Fig 5A-B). Dose-response experiments further confirmed that RGS4 expression increased proportionally with escalating M protein concentrations, establishing a direct dose-dependent regulatory relationship (Fig 5C-D). Furthermore, LD inhibition reversed the M-induced upregulation of RGS4 expression (S3A-B Fig). Given that RGS4 stability is governed by the Cys/N-degron pathway, we investigated whether RABV-M modulates this process by targeting arginyltransferase 1 (ATE1), the key enzyme responsible for substrate arginylation [34]. Cycloheximide-chase assays revealed that ATE1 knockdown significantly attenuated RGS4 degradation rates (Fig 5E-F), confirming ATE1’s role in RGS4 turnover. To validate that the Cys/N-degron pathway is directly targeted by RABV-M, we generated an RGS4 mutant (C2V) with a cysteine-to-valine substitution at the second amino acid position, which abrogates ATE1 recognition [35]. This mutation led to marked RGS4 stabilization. Furthermore, RABV-M overexpression failed to further stabilize this mutant, confirming that RABV-M protects RGS4 from Cys/N-degron-mediated degradation (Fig 5G-H).

**Fig 5:**
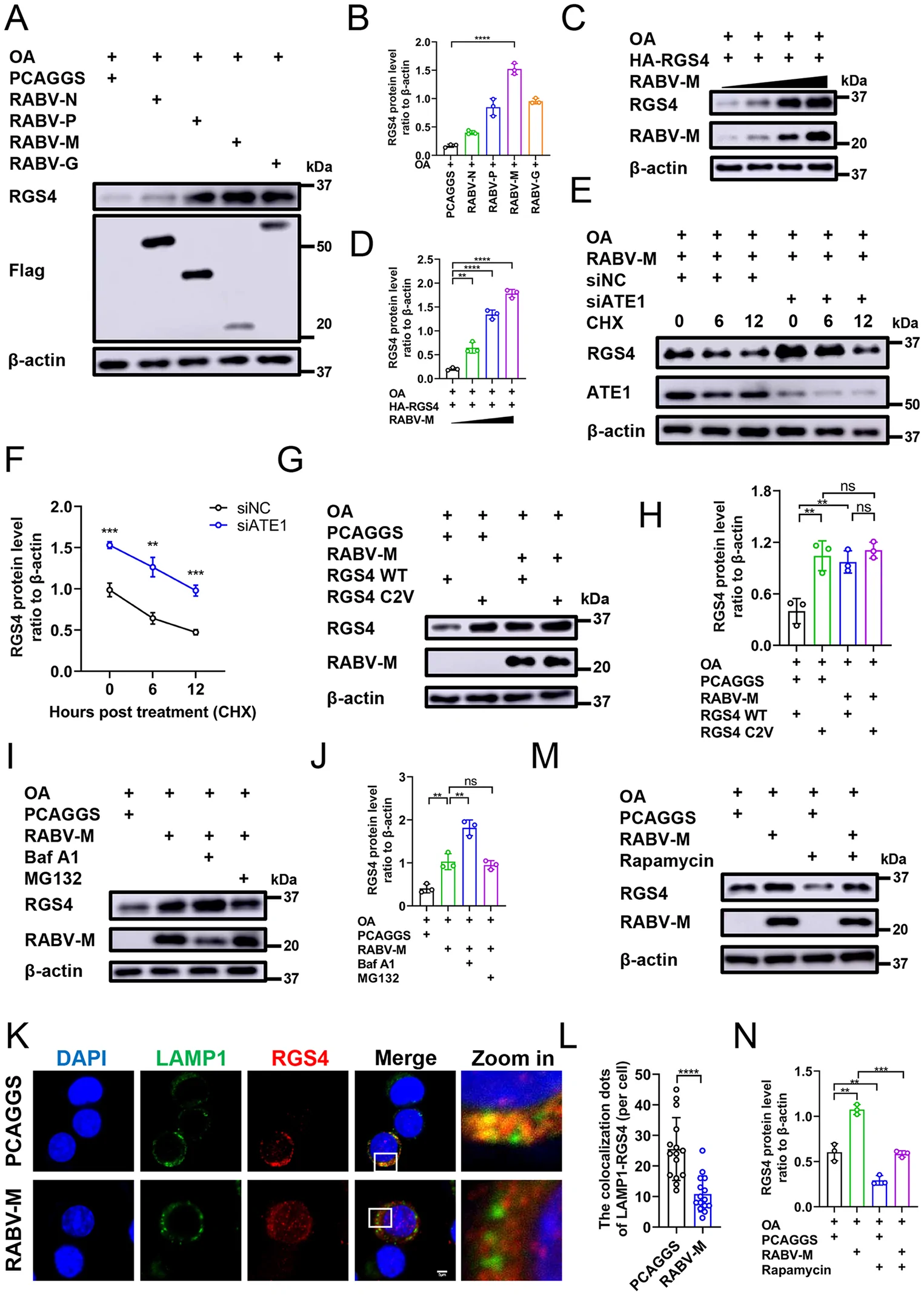
RABV-M induced incomplete autophagy to restrict RGS4 degradation via inhibiting Cys/N-degron pathway. **(A)** Immunoblot analysis and (B) quantification of endogenous RGS4 protein levels in N2a cells treated with oleic acid (OA, 100 μM) and transfected with RABV-N, P, M or **G. (C)** Immunoblot analysis and (D) quantification of exogenous RGS4 protein levels in N2a cells treated with OA and transfected with gradient RABV-M. **(E)** Immunoblot analysis and (F) quantification of endogenous RGS4 protein levels in N2a cells treated with OA and cycloheximide (0.2 mg/mL) and transfected with siATE1. **(G)** Immunoblot analysis and (H) quantification of RGS4 mutation (WT or C2V) protein levels in N2a cells treated with OA and transfected with RABV-M. **(I)** Immunoblot analysis and (J) quantification of endogenous RGS4 proteins level in N2a cells treated with OA, Bafilomycin A1 (10 nM) and MG132 (10 μM). Representative images (K) and co-localization dots (L) of LAMP1 and RGS4 (K, scale bar: 5 μm) in N2a cells treated with OA, rapamycin (1 μM) and transfected with RABV-M or PCAGGS. **(M)** Immunoblot analysis and (N) quantification of endogenous RGS4 protein levels in N2a cells treated with OA, rapamycin and transfected with RABV-M. Error bars represent mean±SD (n = 3). **P < 0.01, ***P < 0.001, ****P < 0.0001, ns, not significant (unpaired two-tailed Student’s t-test or one-way ANOVA).

To identify the specific degradation machinery involved, we treated cells with pharmacological inhibitors of autophagy (Bafilomycin A1) and the proteasome (MG132). BafA1 treatment resulted in significant RGS4 accumulation, while MG132 had no effect (Fig 5I-J), indicating that RGS4 degradation is primarily mediated by autophagy. Since RABV-M is known to induce incomplete autophagy by blocking autophagosome-lysosome fusion [36,37], we hypothesized that M protein impairs RGS4 delivery to lysosomes. Indeed, immunofluorescence analysis showed reduced colocalization of RGS4 with the autolysosome marker LAMP1 in M-transfected cells, even after rapamycin-induced autophagy activation (Fig 5K-L). Consistently, M protein overexpression rescued RGS4 levels in rapamycin-treated cells (Fig 5M-N), confirming that M protein-induced incomplete autophagy prevents RGS4 turnover. Collectively, these data demonstrate that RABV-M stabilizes RGS4 by blocking Cys/N-degron pathway-mediated arginylation and impairing autophagosome-lysosome fusion to prevent lysosomal degradation.

### RABV-M stabilizes RGS4 to drive HERC2-mediated branched K48/K63-linked ubiquitination of NCOA4 and suppress ferroptosis

The HECT and RLD domain-containing E3 ubiquitin ligase HERC2 has been previously shown to regulate NCOA4 expression and subsequent ferritinophagy [38]. To test whether RABV-M modulates this pathway via an RGS4-dependent mechanism, we first assessed HERC2 expression in RGS4 KO cells during RABV infection. Both mRNA and protein levels of HERC2 were significantly downregulated in RGS4 KO cells compared to WT controls (S4A-C Fig). Conversely, RABV-M overexpression upregulated HERC2 while suppressing NCOA4 expression in WT cells, but this effect was completely abrogated in RGS4 KO cells, which exhibited reduced HERC2 and elevated NCOA4 levels (Fig 6A-B). These data establish that RABV-M regulation of the HERC2-NCOA4 axis is strictly dependent on RGS4. To further validate HERC2’s role in NCOA4-dependent ferritinophagy, we performed siRNA-mediated HERC2 knockdown in WT cells. HERC2 silencing restored NCOA4 expression, leading to decreased FTH levels and a marked accumulation of labile ferrous iron (Fe^2+^) (Fig 6C-F). Given that NCOA4 stability is dictated by the ubiquitin-proteasome system [38,39], we treated cells with the proteasome inhibitor MG132. HERC2 knockdown significantly increased NCOA4 levels, and MG132 treatment resulted in further stabilization (Fig 6G-H), confirming that HERC2 promotes NCOA4 degradation via the proteasome. To map the specific ubiquitin linkage involved in NCOA4 turnover, we co-expressed RABV-M with K63-only or K48-only ubiquitin mutants. Our results demonstrated that the RGS4-HERC2 axis specifically regulates NCOA4 stability through K63-linked ubiquitination (Fig 6I). Furthermore, when K63 and K48 were individually mutated, the ubiquitination levels of NCOA4 were significantly reduced in both cases, indicating that NCOA4 stability is regulated by K48/K63 branched ubiquitin chains (Fig 6J). Crucially, K63-linked ubiquitination of NCOA4 was significantly attenuated in RGS4 KO cells (Fig 6K), underscoring the essential role of RGS4 in facilitating HERC2-mediated ubiquitination. Collectively, these findings delineate a complete regulatory axis: RABV-M stabilizes RGS4 to maintain HERC2 expression, which in turn drives branched K48/K63-linked ubiquitination of NCOA4, suppressing ferritinophagy and limiting cellular susceptibility to ferroptosis to promote viral replication.

**Fig 6:**
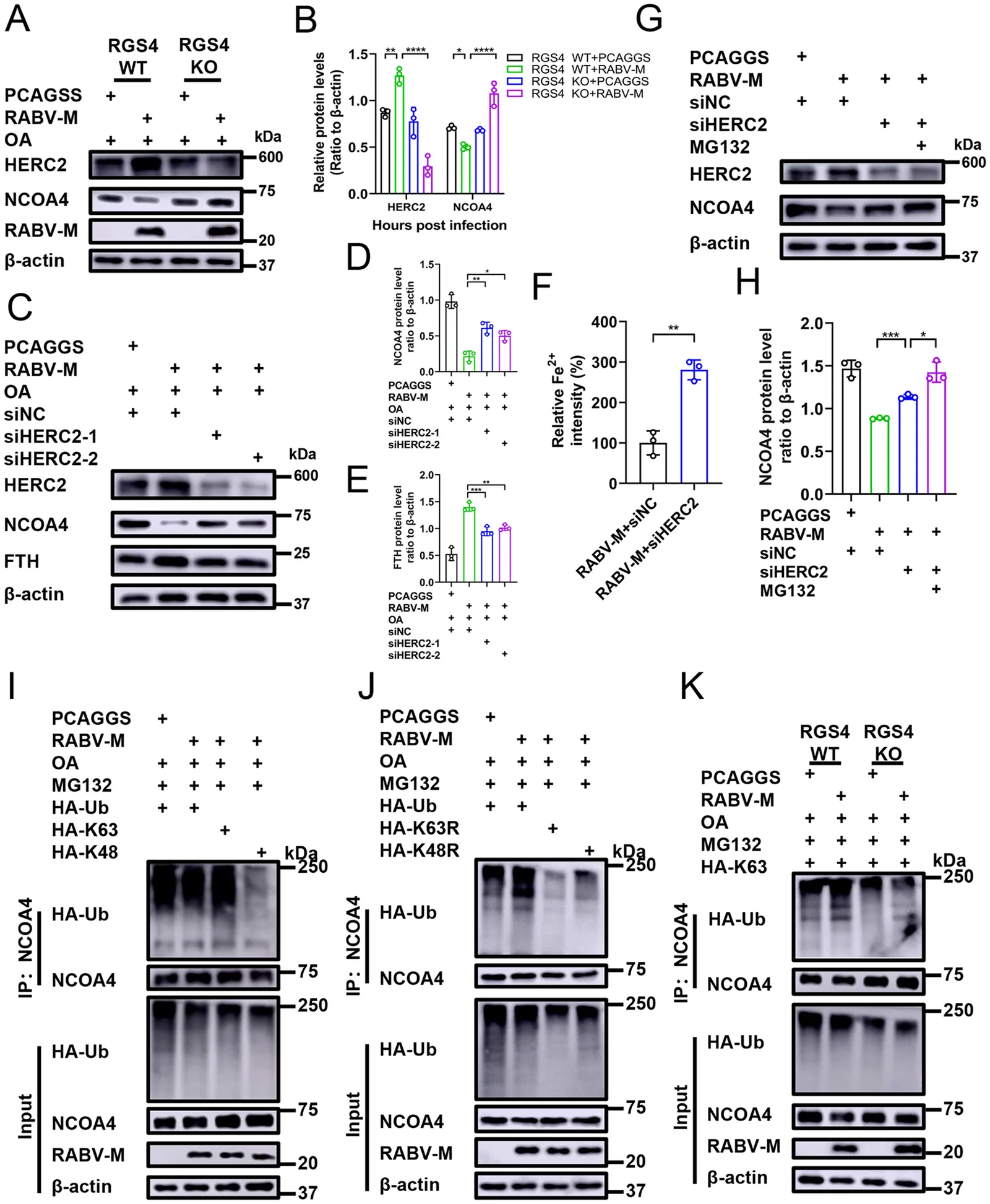
RABV M protein modulated ferritinophagy through restricting HERC2 mediated ubiquitination via maintaining RGS4 stability. **(A)** Immunoblot analysis and (B) quantification of HERC2 and NCOA4 protein levels in RGS4 WT or RGS4 KO N2a cells treated with OA (100 μM) and transfected with RABV-M. **(C)** Immunoblot analysis and quantification of NCOA4 (D) and FTH (E) protein levels in N2a cells treated with OA and transfected with RABV-M and siHERC2. **(F)** Quantification of Fe^2+^ in N2a cells treated with OA and transfected with RABV-M and siHERC2. **(G)** Immunoblot analysis and (H) quantification of NCOA4 protein levels in N2a cells treated with OA, MG132 (10 μM) and transfected with RABV-M, siHERC2. **(I)** Co-IP analysis of HA-Ub (HA-Ub-K63 or HA-Ub-K48) and NCOA4 in N2a cells treated with OA, MG132 and transfected with indicated plasmids. **(J)** Co-IP analysis of HA-Ub (HA-Ub-K63R or HA-Ub-K48R) and NCOA4 in N2a cells treated with OA, MG132 and transfected with indicated plasmids. **(K)** Co-IP analysis of HA-Ub-K63 and NCOA4 in RGS4 WT or RGS4 KO N2a cells treated with OA, MG132 (10 μM) and transfected with indicated plasmids. Error bars represent mean±SD (n = 3). *P < 0.05, **P < 0.01, ***P < 0.001, ****P < 0.0001, ns, not significant (unpaired two-tailed Student’s t-test or one-way ANOVA).

### Lyssavirus M proteins conserve the RGS4-HERC2-NCOA4 axis to inhibit ferritinophagy and ferroptosis

To determine whether the M protein-mediated regulation of ferroptosis via RGS4 is evolutionarily conserved across the lyssavirus genus, we analyzed M proteins from two phylogenetically distinct strains: a street isolate of dog-derived rabies virus (DRV) and Australian bat lyssavirus (ABLV). Consistent with our observations for prototype RABV-M, both DRV-M and ABLV-M significantly upregulated HERC2 and FTH expression while downregulating NCOA4, thereby inhibiting ferritinophagy (Fig 7A-B). HERC2 knockdown reversed this protective phenotype, leading to a marked accumulation of labile ferrous iron (Fe^2+^) (Fig 7C), confirming that suppression of NCOA4-mediated FTH degradation is strictly dependent on HERC2 across lyssaviruses. To validate that lyssavirus M proteins stabilize RGS4 by interfering with the Cys/N-degron pathway, we introduced a cysteine-to-valine substitution (C2V) at the second amino acid position of RGS4- a mutation that confers resistance to ATE1-mediated arginylation and degradation. Expression of the RGS4 C2V mutant resulted in significantly higher steady-state RGS4 levels in cells transfected with either DRV-M or ABLV-M (Fig 7D-G), confirming that these viral proteins share the same mechanism of RGS4 stabilization as RABV-M. Finally, ubiquitination assays demonstrated that DRV-M and ABLV-M promote HERC2-dependent, branched K48/K63-linked ubiquitination of NCOA4 in an RGS4-dependent manner. This modification was significantly attenuated in RGS4 KO cells (Fig 7H-I), underscoring the essential role of RGS4 in facilitating HERC2 activity across lyssaviruses. Collectively, these findings establish that stabilization of RGS4 to suppress NCOA4-mediated ferritinophagy and modulate ferroptosis susceptibility is a conserved immune evasion strategy employed by diverse members of the lyssavirus genus to promote viral replication (Fig 8).

**Fig 7:**
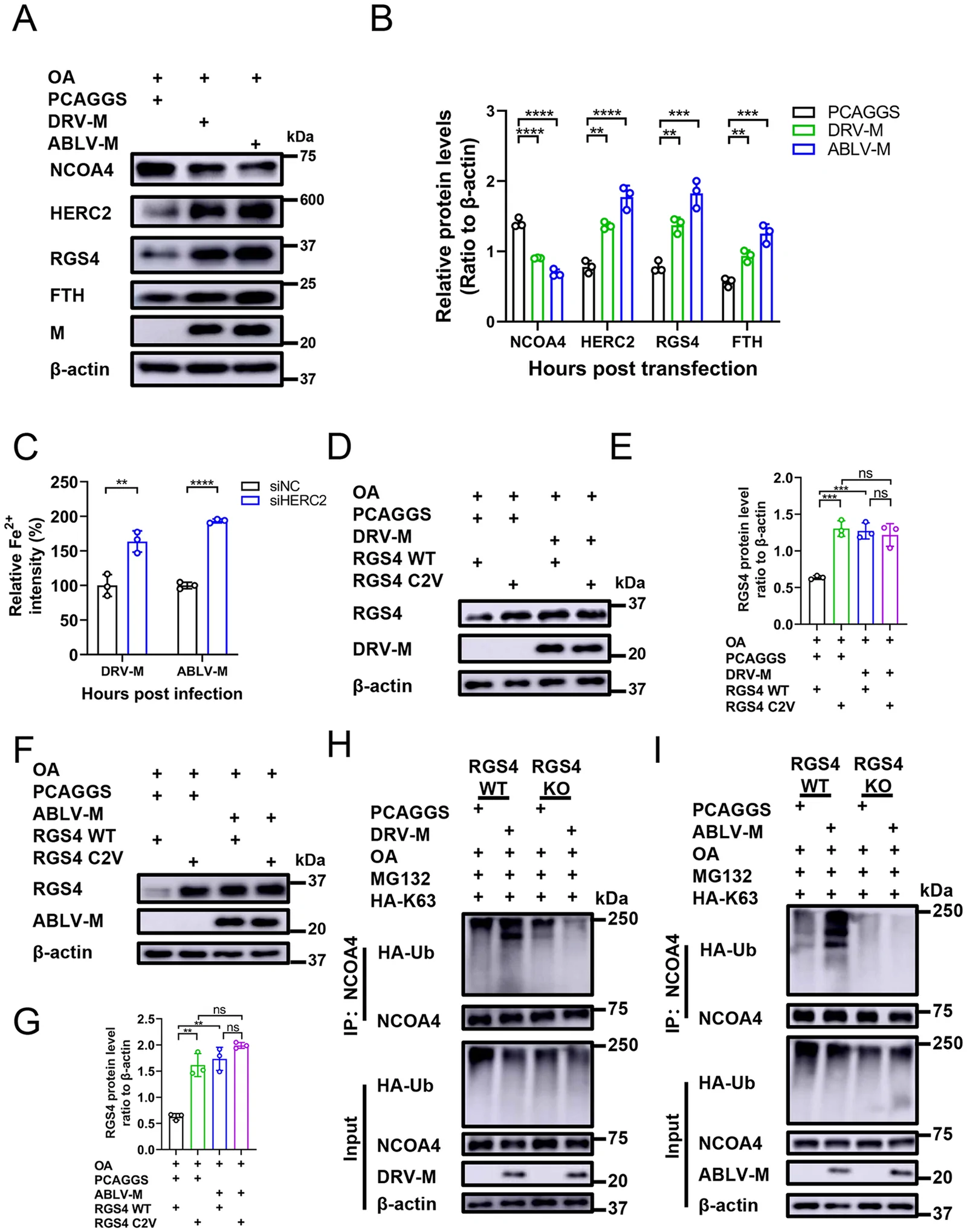
Lyssavirus M protein maintained RGS4 stability to modulate NCOA4 mediated ferritinophagy to regulate ferroptosis susceptibility. **(A)** Immunoblot analysis and (B) quantification of HERC2, NCOA4, RGS4 and FTH protein levels in N2a cells treated with OA (100 μM) and transfected with DRV-M or ABLV-M. **(C)** Quantification of Fe^2+^ in N2a cells treated with OA and transfected with DRV-M (or ABLV-M) and siHERC2. **(D)** Immunoblot analysis and (E) quantification of RGS4 mutation (WT or C2V) protein levels in N2a cells treated with OA and transfected with DRV-M. **(F)** Immunoblot analysis and (G) quantification of RGS4 mutation (WT or C2V) protein levels in N2a cells treated with OA and transfected with ABLV-M. Co-IP analysis of HA-Ub-K63 and NCOA4 in N2a cells treated with OA, MG132 (10 μM) and transfected with DRV-M (H) or ABLV-M **(I)**. Error bars represent mean±SD (n = 3). **P < 0.01, ***P < 0.001, ****P < 0.0001, ns, not significant (unpaired two-tailed Student’s t-test or one-way ANOVA).

**Fig 8:**
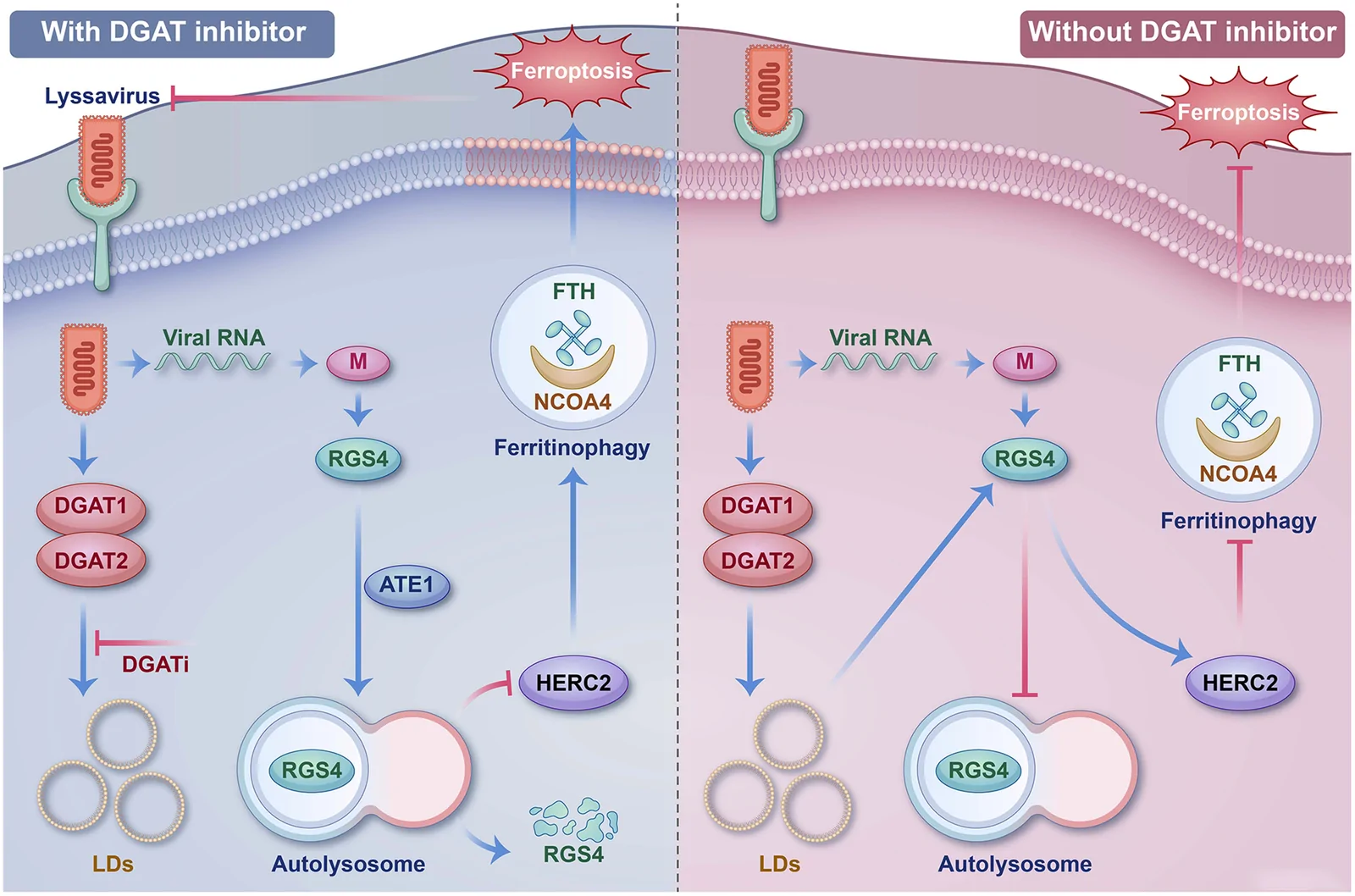
Schematic model of Lyssavirus M protein-mediated ferroptosis resistance via stabilizing RGS4 to modulate NCOA4-mediated ferritinophagy. Upon lyssavirus infection, DGAT-mediated LD formation and matrix protein cooperate to suppress RGS4 degradation through an ATE1-mediated autophagic pathway. This leads to increased HERC2 expression, which ultimately restricts NCOA4-mediated ferritinophagy and reduces ferroptosis susceptibility to promote viral replication. Inhibiting DGAT to reduce LD formation in lyssavirus infection induces RGS4 degradation via ATE1 mediated autophagic pathway, which further promotes NCOA4 mediated ferritinophagy to enhance ferroptosis susceptibility to restrict viral replication.

## Discussion

Ferroptosis plays a complex, context-dependent role in viral infections, serving as both a host defense mechanism to restrict pathogen replication and a viral strategy to promote dissemination [40–42]. Our previous work established that lyssavirus-induced LD biogenesis serves a dual function: it provides structural platforms for viral budding and suppresses arachidonic acid-mediated ferroptosis by sequestering arachidonic acid into neutral lipids, thereby reducing its availability for peroxidation and promoting viral replication [5,17]. Building on this foundation, the current study unveils a second, parallel mechanism by which LDs enhance viral replication: LD formation stabilizes the host protein RGS4, which in turn maintains HERC2-mediated branched K48/K63-linked ubiquitination of NCOA4, inhibiting ferritinophagy and subsequent ferroptosis induction. Although our data are most consistent with branched K48/K63 architectures on NCOA4, definitive proof of chain branching will require mass-spectrometric detection of doubly modified ubiquitin peptides; nonetheless, branched chains of this class have been well documented to convey enhanced proteasomal degradation signals [43,44]. This discovery expands our understanding of lyssavirus pathogenesis, revealing how the virus co-opts host lipid and iron metabolism pathways in a coordinated manner to suppress ferroptosis and ensure viral survival. Furthermore, regarding the mechanism by which the M protein regulates HERC2 expression, we hypothesize that the M protein, via RGS4, sustains HERC2 expression at the transcriptional and/or post-transcriptional level. NRF2 has been reported to bind to the promoter sequence of HERC2 and promote its transcription [45,46]. Additionally, as a member of the E3 ubiquitin ligase family, HERC2 protein stability also may be regulated by the ubiquitin-proteasome pathway. The precise mechanism by which lyssavirus M protein regulates HERC2 at these multiple levels remains to be fully elucidated. Collectively, these findings illustrate that lyssavirus employs a multi-layered strategy to modulate ferroptosis: first, by redirecting arachidonic acid into LDs to limit substrate availability for lipid peroxidation; second, by stabilizing RGS4 to inhibit ferritinophagy and iron-dependent lipid peroxidation. This dual suppression of ferroptosis not only prevents premature cell death but also reduces the release of damage-associated molecular patterns (DAMPs) from dying cells, thereby attenuating CNS inflammation. This aligns with the relatively low inflammatory response observed in natural rabies infections [47], highlighting how lyssavirus has evolved to replicate efficiently while minimizing immune activation.

Notably, the role of ferroptosis in viral infections is highly context-dependent, with different viruses exploiting this pathway in distinct ways to promote replication. For example, Influenza A virus (IAV) utilizes its hemagglutinin protein to interact with NCOA4, inducing ferritinophagy and ferroptosis to enhance viral spread [48]. Similarly, pharmacological inhibition of ACSL1-mediated ferroptosis reduces murine hepatitis virus (MHV) infection [49]. Moreover, ACSL4-dependent ferroptosis promotes replication of enteroviruses and coronaviruses, NCOA4-dependent ferritinophagy facilitates Pseudorabies virus (PRV) replication and Monkeypox virus (MPXV) upregulates prostaglandin-endoperoxide synthase 2 (PTGS2) to induce ferroptosis for viral replication [50–52]. These examples highlight a growing recognition of ferroptosis as a critical node in host-virus interactions, with viruses either inducing or inhibiting this pathway based on their specific replication requirements. Furthermore, ferroptosis also serves as a critical host defense mechanism to restrict viral replication. For instance, the ferroptosis agonist RSL3 has been shown to activate ferroptosis and thereby limit PEDV replication [53]. Zinc ions can induce ferroptosis via the xCT/GPX4 axis to suppress the replication of Infectious Spleen and Kidney Necrosis Virus (ISKNV) [54]. Additionally, HCV replication is highly sensitive to intracellular lipid peroxidation levels [55]. Fatty Acid Desaturase 2 (FADS2) restricts HCV replication by converting oleic acid into highly unsaturated fatty acids, which are prone to peroxidation. Moreover, the ferroptosis inducer Erastin has also been found to restrict HCV replication by inducing conformational changes in the HCV replicase [56]. In contrast to viruses that induce ferroptosis, lyssavirus has evolved to suppress this pathway, likely due to its unique neurotropic lifestyle. Unlike respiratory viruses that rely on widespread cell death for transmission, lyssavirus requires long-term neuronal survival to travel from the site of infection to the CNS and establish productive replication. By suppressing ferroptosis, the virus ensures that infected neurons remain viable, allowing for efficient viral spread and dissemination. This distinction underscores the remarkable adaptability of viruses to exploit host pathways in a manner that aligns with their specific life cycle requirements. Furthermore, viral infection can induce various forms of cell death [57–59], while simultaneously promoting the production of multiple cytokines and causing tissue damage [60,61]. This suggests a potential interplay between different cell death pathways. Previous studies have found that ferroptosis metabolites can antagonize necroptosis [62]. Given that RABV infection can also induce other forms of cell death, such as apoptosis and pyroptosis [63,64], and modulates immune responses through the assembly of inflammasomes [65], we hypothesize that RABV may regulate immune responses and multiple cell death pathways in a coordinated manner, ultimately influencing cell fate. This intricate interplay warrants further investigation in the future.

Incomplete autophagy mediated by the M protein has been established to facilitate lyssavirus budding and proliferation through autophagic vesicles by inhibiting autolysosomal degradation [36,37,66]. In this study, we further elucidate an additional mechanistic pathway through which M-induced incomplete autophagy promotes viral replication: incomplete autophagy effectively prevents FTH degradation within lysosomes, thereby restricting intracellular iron release and reducing ferroptosis susceptibility. Furthermore, the M protein orchestrates iron homeostasis by stabilizing RGS4, which in turn maintains HERC2-dependent ubiquitination of NCOA4, attenuating NCOA4’s role as a selective cargo receptor for ferritinophagy. Notably, viral protein-induced incomplete autophagy exhibits mechanistic diversity in facilitating viral replication. For instance, the gM protein of pseudorabies virus (PRV) suppresses autophagosome-lysosome fusion by caspase 3 (CASP3)-mediated SNAP29 degradation, thereby enhancing viral replication [67]. Southern rice black-streaked dwarf virus (SRBSDV) employs its capsid protein P10 to interact with LAMP1, blocking autophagosome-lysosome fusion and evading degradation [68]. Rice gall dwarf virus (RGDV) utilizes its P2 protein to directly recruit the GAPDH-ATG4B complex for nascent autophagosome formation while inhibiting ATG14-SNARE-mediated autophagosome-lysosome fusion, promoting viral persistence [69]. Furthermore, Bovine parainfluenza type 3 virus (BPIV3), Hepatitis B surface antigen (HBsAg) and SARS-CoV-2 ORF7a can also induce incomplete autophagy for viral replication [70–72]. Notably, current research predominantly emphasizes the role of virus-induced incomplete autophagy in viral particle assembly and budding, with limited exploration of its impact on cellular fate regulation to support viral replication. Our findings establish a paradigm-shifting mechanism wherein incomplete autophagy exerts dual functions: not only facilitating virion morphogenesis but also modulating cellular fate through iron metabolic reprogramming to create a replication-permissive environment, thereby uncovering a novel dimension of host-virus interactions.

RGS4 represents a canonical substrate of the Cys/N-degron pathway, which undergoes degradation via either ATE1-mediated proteasomal activity or autophagy [73–75]. Notably, this Cys/N-degron pathway differentially employs proteasomal degradation under normoxic conditions and autophagy under hypoxic stress to eliminate RGS4 [34,35]. In the present study, we demonstrate that lyssavirus M stabilizes RGS4 by specifically restricting its autophagic degradation. Previous studies have established that RABV M protein induces mitochondrial damage and subsequent apoptosis [76], mitochondrial impairment consequentially enhances reactive oxygen species (ROS) production [77]. This apparent paradox reveals that although the M protein triggers ROS generation, its induction of incomplete autophagy also counteracts oxidative stress signaling by preserving RGS4 stability, thereby modulating ferroptosis susceptibility. Intriguingly, this mechanism aligns with our observation that virus-induced LD biogenesis may mitigate mitochondrial damage and subsequent ROS accumulation. For instance, expression of the viral nucleoprotein effectively counteracts apoptosis induction [76], suggesting a coordinated strategy where LD biogenesis mitigates oxidative damage while incomplete autophagy synergistically stabilizes RGS4 to modulate cellular redox homeostasis during infection. Although the M protein plays the predominant role in stabilizing RGS4, our data also show a modest stabilizing effect exerted by N, P, and G proteins. This raises the possibility of a synergistic interaction among viral proteins that collectively reinforces the inhibition of ferroptosis.

In addition, RGS4 has been established as a signature gene associated with ferroptosis susceptibility [28,78–80], though the precise mechanisms through which RGS4 regulates ferroptosis remain incompletely defined. Studies have demonstrated that RGS4 modulates glutamatergic dysfunction through SLC7A11 regulation, leading to schizophrenia-like behavioral phenotypes [30]. Given that SLC7A11 serves as a key component of the SLC7A11/GSH/GPX4 axis [81,82], these observations suggest a potential regulatory role for RGS4 in antioxidant defense systems. Our findings reveal a novel mechanism by which RGS4 modulates ferroptosis susceptibility through iron metabolic regulation.

In summary, our study identifies a novel regulatory axis by which lyssavirus modulates host iron metabolism to suppress ferroptosis, contributing to the growing understanding of the diverse roles of ferroptosis in viral infections. These findings not only advance our understanding of rabies pathogenesis but also provide a novel strategy for investigating lyssavirus replication through the lens of metabolism and ferroptosis.

## Methods

### Ethics statement

The animal experiments were carried out strictly according to the protocols and approved by the Scientific Ethics Committee of Huazhong Agricultural University (permit number: 202412260012). All institutional and national guidelines for the care and use of laboratory animals were followed.

### Animals, cells, antibodies, chemical inhibitors and regents

N2a (CCL-131) cell line were obtained from American Type Culture Collection (ATCC). Primary neurons were isolated and cultured as previous described [83]. Dulbecco’s medium (DMEM) and fetal bovine serum (FBS) were purchased from Gibco. An anti-β-actin antibody (AC026), an anti-FTH1 antibody (A25458PM), an anti-HA antibody (AE105), an anti-LC3B antibody (A19665), an anti-Flag antibody (AE005), and an ABflo 488 anti-LAMP1 antibody (A24362) were purchased from ABclonal (Wuhan, China). An anti-RGS4 antibody (66441–1-lg) was purchased from Proteintech (Wuhan, China). An anti-NCOA4 antibody (ab314553) were purchased from Abcam. An HRP-conjugated goat anti-mouse antibody (BA1051) and a goat anti-rabbit antibody (BA1055) purchased from Boster (Wuhan, China). An Alexa Fluor plus 594 goat anti-rabbit IgG (H + L) cross-adsorbed secondary antibody (A32740) was purchased from Invitrogen. Oleic acid (T2O2668), cycloheximide (T1225), MG132 (T2154), Chloroquine (T8689), and Bafilomycin A1 (T6740) were purchased from TargetMol (MA, USA). MDA Assay Kit (M496), BDP 581/591 C11 (L267), FerroOrange (F374) and GSSG/GSH Quantification Kit II (L263) were purchased from DoJindo. Hydrogen Peroxide Assay Kit (S0038), Penicillin-streptomycin (C0222), PI (ST511) and Western/IP lysis buffer (C0013) were obtained from Beyotime (Shanghai, China). HiScript II 1st Strand cDNA Synthesis Kit (R211-01), HiScript III 1st Strand cDNA Synthesis Kit (R222-01) and ChamQ SYBR qPCR Master Mix (Q711-02) were purchased from Vazyme (Nanjing, China). Prolong Gold antifade mounting solution (P10144) was purchased from Invitrogen.

### Reduced glutathione (GSH) and oxidized glutathione disulfide (GSSG) measurement

RGS4 WT or RGS4 KO N2a cells were infected with RABV (MOI = 0.1, 48 h). Cells were subsequently harvested, washed with ice-cold phosphate-buffered saline (PBS), and lysed. Cellular reduced glutathione (GSH) and oxidized glutathione (GSSG) concentrations were quantified using the GSSG/GSH Quantification Kit II (Dojindo) according to the manufacturer’s protocol. Absorbance was measured at 405 nm using a SpectraMax 190 microplate reader (Molecular Devices, CA). GSH and GSSG levels were calculated by interpolation from respective standard curves.

### RNA-seq analysis

Total RNA was extracted using TRIzol reagent as the manufacturer’s instructions. Stranded RNA-seq libraries were prepared on the BGI sequencing platform in accordance with the provided protocol. RNA-seq reads were mapped to the GRCm39.112 reference genome assembly. Differential gene expression analysis was performed using DESeq2 (v1.26.0), with statistical significance determined by Benjamini-Hochberg-corrected P values to control for false discovery rate (FDR).

### Quantitative PCR analysis

Total RNA was isolated using TRIzol reagent following the manufacturer’s protocol. Purified RNA was reverse-transcribed into cDNA using the HiScript III 1st Strand cDNA Synthesis Kit. Quantitative PCR was performed on a QuantStudio 7 Real-Time PCR System (Applied Biosystems, USA) with the following thermal cycling parameters: initial denaturation at 95 °C for 2 minutes, followed by 40 cycles of 95 °C for 5 seconds and 60 °C for 30 seconds. Gene expression levels were quantified relative to an endogenous control gene. Primer sequences are listed in S1 Table of the Supporting Information.

### Western blotting assay

Cells were lysed in Western/IP lysis buffer and centrifuged at 12,000 g, 4 °C. Supernatants were denatured in SDS loading buffer, resolved by SDS-PAGE, and transferred to PVDF membranes. After blocking with 5% skim milk for 1 h, membranes were incubated with primary antibodies overnight at 4 °C, washed three times with TBST, and then probed with species-specific secondary antibodies for 1 h at room temperature. Protein signals were detected using an Amersham Imager 600 (GE Healthcare).

### Cell death, lipid peroxidation assay and Fe^2+^ measurement

Propidium iodide (Ex: 535 nm, Em: 617 nm), Probes of BDP 581/591 C11 (non-oxidized, Ex: 550 nm, Em: 590 nm; oxidized,Ex: 480 nm, Em: 530 nm), FerroOrange (Ex: 543 nm, Em: 580 nm) were used to determined cell death, lipid peroxidation, Fe^2+^,respectively. RGS4 WT or RGS4 KO N2a cells were transfected with shNCOA4 (three short harpin of RNAs were provided in S1 Table) and infected with RABV (MOI = 0.1, 48 h). Cells were harvested, washed with ice-cold phosphate-buffered saline (PBS), and incubated with the respective detection probes: Propidium iodide, BDP 581/591 C11 or FerroOrange. Cells were stained for 30 minutes at 37 °C. Following PBS washing for twice, cell suspensions were counted using a Countstar automated cell counter. 10⁴ cells per sample were analyzed using a fluorescent microplate reader (Tecan).

### RGS4 knockout N2a cell line construction

Three sgRNAs targeting RGS4 were designed, including RGS4-sgRNA-1 (5’-TGTGCCTAATTCTCACACAGAGG-3’), RGS4-sgRNA-2 (5’-TGACTTCTGGATCAGCT

GTGAGG-3’), and RGS4-sgRNA-3 (5’-CAGTTCTTCACACAGCAAGAAGG-3’), and subcloned into the px458-Cas9-puro vector. Recombinant plasmids were transfected into N2a cells, isolated transfection-competent clones via puromycin, and expanded in culture for subsequent functional assays.

### Confocal microscopy

RGS4 WT or RGS4 KO N2a cells were seeded on poly-L-lysine-coated coverslips and infected with RABV (MOI = 0.1) for 48 h post-seeding. After fixation with 4% paraformaldehyde at room temperature (RT) for 30 min, cells were quenched with 0.1 M glycine in PBS (15 min), followed by three PBS washes. Permeabilization was achieved using 0.1% Triton X-100 in PBS (30 min). Following blocking with 10 mM Tris-HCl (pH 7.5), 150 mM NaCl, 2% BSA, and 10% goat serum (30 min), cells were incubated with primary antibodies (anti-LAMP1, anti-FTH1) for 2 h. After PBS washing, cells were incubated with Alexa Fluor-conjugated secondary antibodies (488/594) for 2 h at RT. Nuclei were counterstained with DAPI (5 min), and coverslips were mounted in ProLong Gold Antifade Mountant. Images were acquired using a Nikon confocal laser scanning microscope with excitation lasers at 405 nm (DAPI), 488 nm (Alexa Fluor 488) and 561 nm (Alexa Fluor 594).

### Co-immunoprecipitation (Co-IP)

Cultured cells were washed with ice-cold PBS and lysed in Western/IP buffer supplemented with protease and phosphatase inhibitor cocktails (1×) for 45 min at 4 °C with gentle rotation. Lysates were centrifuged at 12,000 g, 4 °C for 10 min, and supernatants collected. Protein A/G magnetic beads were pre-incubated with target-specific primary antibodies or species-matched IgG isotype controls at RT for 45 min with gentle rotation. After three washes in lysis buffer, antibody-bound beads were incubated with supernatants overnight at 4 °C. Immunocomplex-bound beads underwent five additional lysis buffer washes, resuspension in SDS loading buffer, and denaturation at 95 °C for 5 min. Eluted proteins were separated by SDS-PAGE for immunoblot analysis.

### Malonaldehyde (MDA) assay

RGS4 WT or RGS4 KO N2a cells were infected with RABV (MOI = 0.1) for 48 h. Cells were harvested, washed with ice-cold PBS, and counted using a Countstar automated cell counter. Lysates were processed for malondialdehyde (MDA) quantification per manufacturer’s protocol. TBA-MDA adducts were formed and measured via fluorescent microplate reader (Tecan; Ex: 540 nm, Em: 590 nm).

### Statistical analysis

Statistical analysis was performed using GraphPad Prism 9 (GraphPad Software, San Diego, CA). Immunoblot band intensities and mean fluorescence intensity (MFI) were quantified with ImageJ (https://imagej.nih.gov/ij/). Data are representative of three independent experiments. Statistical significance was assessed using unpaired two-tailed t-tests or one-way ANOVA, with significance denoted as: *, *P* < 0.05; **, *P* < 0.01; ***, *P* < 0.001, ****, *P* < 0.0001, ns, not significant.

## Supporting information

S1 Fig Inhibiting RGS4 activity by small molecule inhibitor CCG-63802 restricts RABV production. (A) Viral titers in N2a cells treated with CCG-63802 (10 μM) and infected with RABV (MOI = 0.1 or 1, 24 h). (B) Quantification of viral genome RNA in N2a cells treated with CCG-63802 and infected with RABV (MOI = 0.1 or 1, 24 h). Error bars represent mean±SD (n = 3). **P < 0.01, ***P < 0.001 (unpaired two-tailed Student’s t-test).
(TIF)

S2 Fig RGS4 knockdown also increased ferroptosis susceptibility in BSR cells. (A) Quantification of cell death in siRGS4 transfected BSR cells treated with Ferrostatin-1 (10 μM), Z-VAD-FMK (25 μM) and GSK872 (2.5 μM) and infected with RABV (MOI = 0.1, 48 h). (B) Viral titers in siRGS4 transfected BSR cells treated with Ferrostatin-1 (10 μM) and infected with RABV. (C) Immunoblot analysis and (D) quantification of RABV-N protein levels in siRGS4 transfected BSR cells treated with Ferrostatin-1 and infected with RABV. Error bars represent mean±SD (n = 3). **P < 0.01, ***P < 0.001, ****P < 0.0001 (unpaired two-tailed Student’s t-test).
(TIF)

S3 Fig LD inhibition abrogated RABV M-mediated RGS4 stabilization. (A) Immunoblot analysis and (B) quantification of exogenous RGS4 protein levels in N2a cells treated with OA (100 μM), DGAT inhibitors (A922500, 8 μg/mL; PF06424439, 10 μg/mL) and transfected with RABV-M. Error bars represent mean±SD (n = 3). ***P < 0.001 (unpaired two-tailed Student’s t-test).
(TIF)

S4 Fig Knocking out RGS4 significantly decreased HERC2 expression. (A) Quantification of HERC2 mRNA level in RGS4 WT and RGS4 KO N2a cells infected with RABV (MOI = 0.1, 48 h). (B) Immunoblot analysis and (C) quantification HERC2 protein levels in RGS4 WT and RGS4 KO N2a cells infected with RABV. Error bars represent mean±SD (n = 3). ***P < 0.001 (unpaired two-tailed Student’s t-test).
(TIF)

S1 Table A. Primers for qPCR (*mus musculus*). B. Primers for qPCR (virus). C. Sequences for shRNA/siRNA. (XLSX)

S1 Raw Gel Rawgel. (PDF)
