# Supplementary material for: Lyssavirus matrix protein stabilizes RGS4 to suppress ferroptosis via incomplete autophagy and iron metabolic reprogramming
Source: PLoS Pathog. 2026 Oct 21;22(10):e1014726. doi: 10.1371/journal.ppat.1014726 (PMC13712672; doi:10.1371/journal.ppat.1014726)

Figure 1D

RGS4

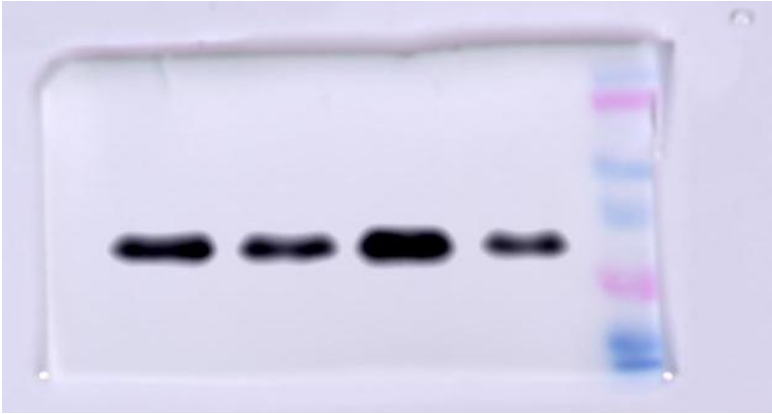

RABV-N

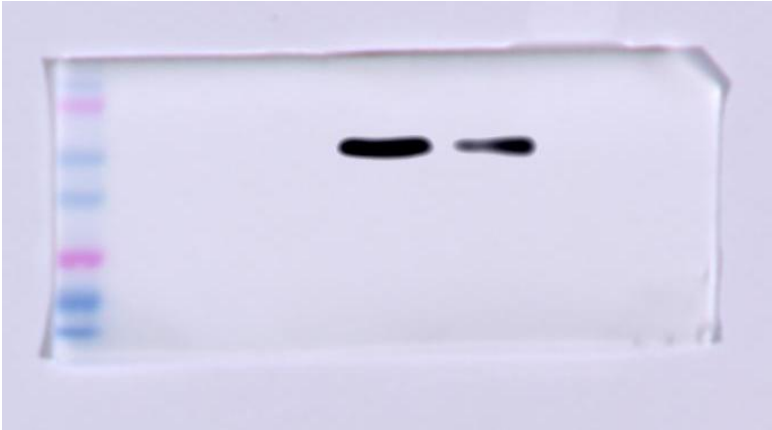

β-actin

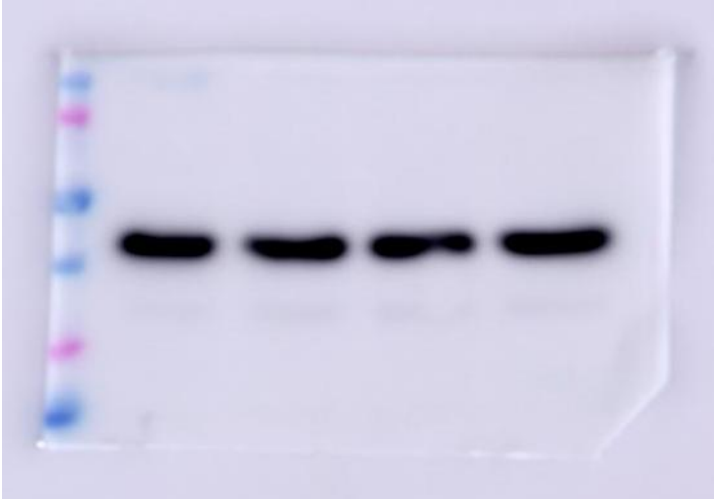

Figure 1F

RGS4

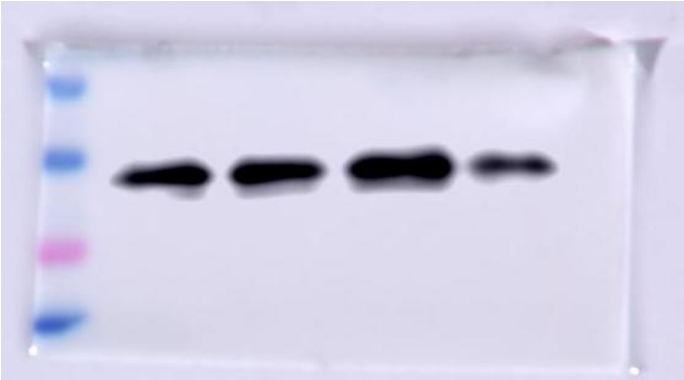

RABV-N

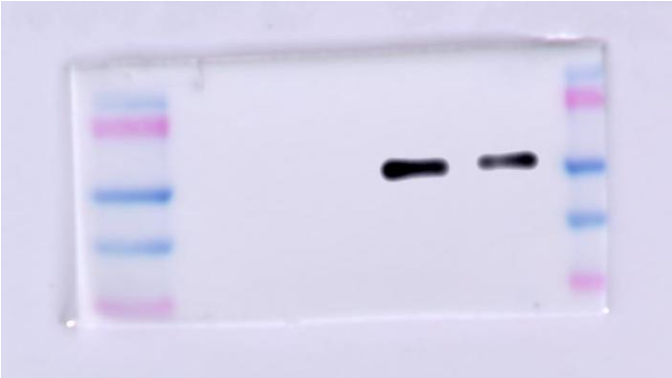

β-actin

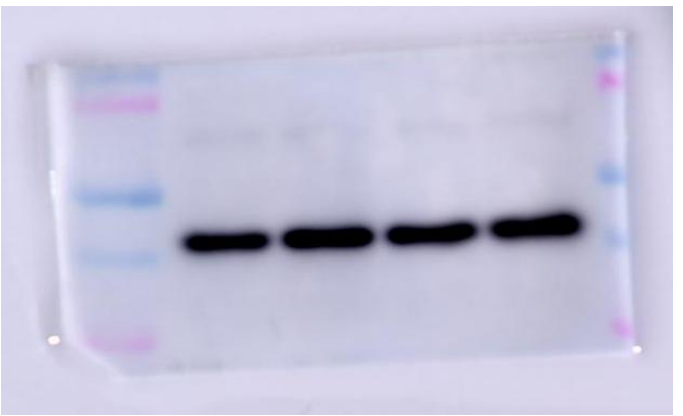

Figure 2B

RGS4

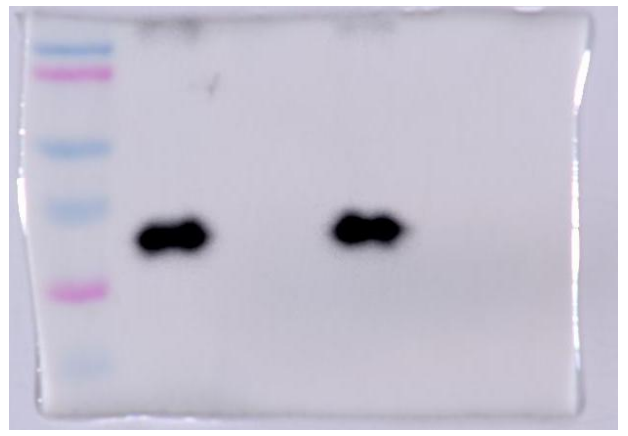

RABV-N

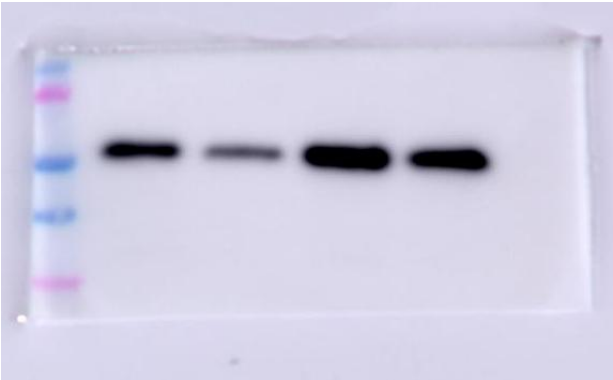

β-actin

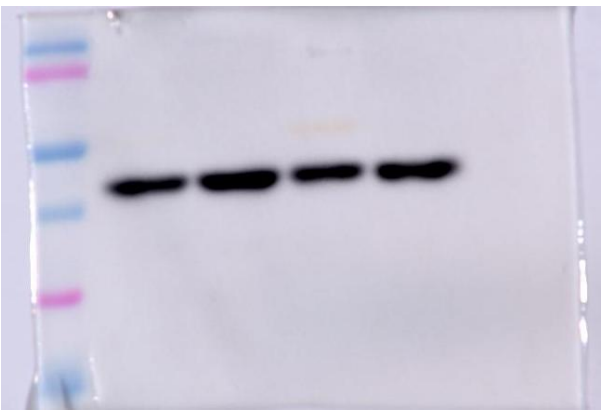

Figure 3L

RABV-N

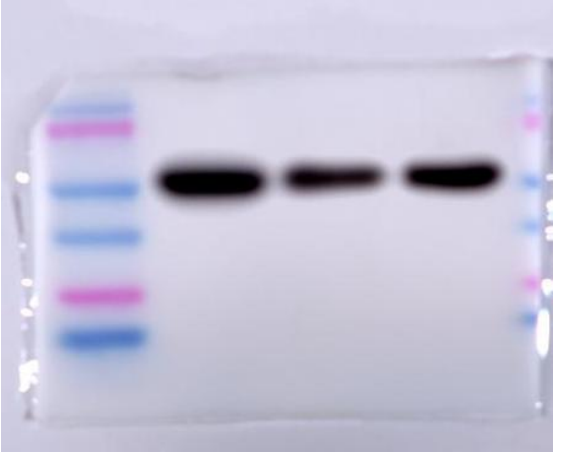

β-actin

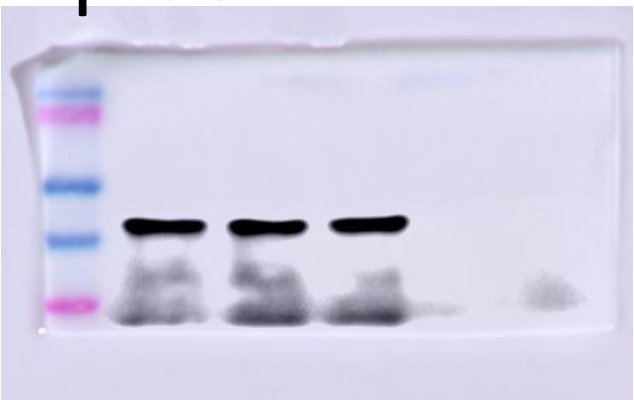

Figure S2

RABV-N

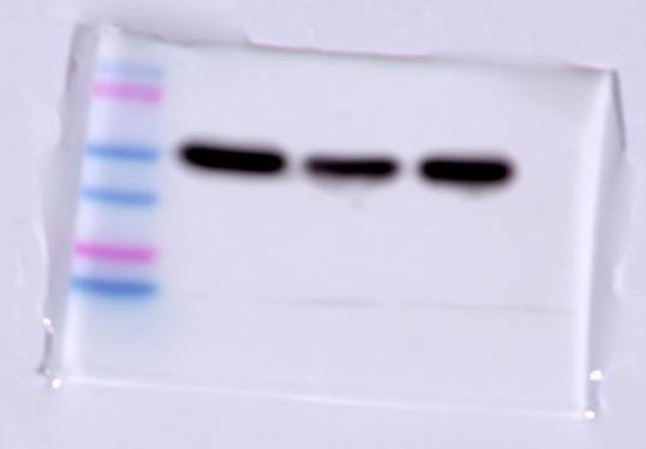

RGS4

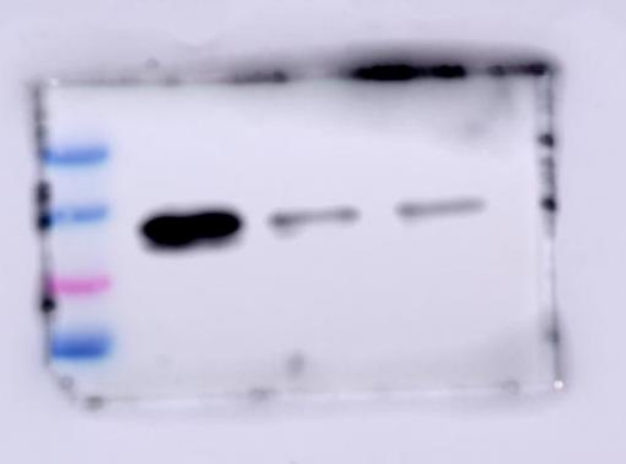

β-actin

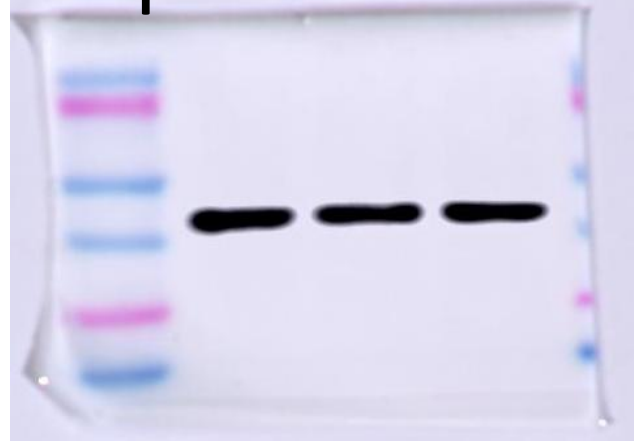

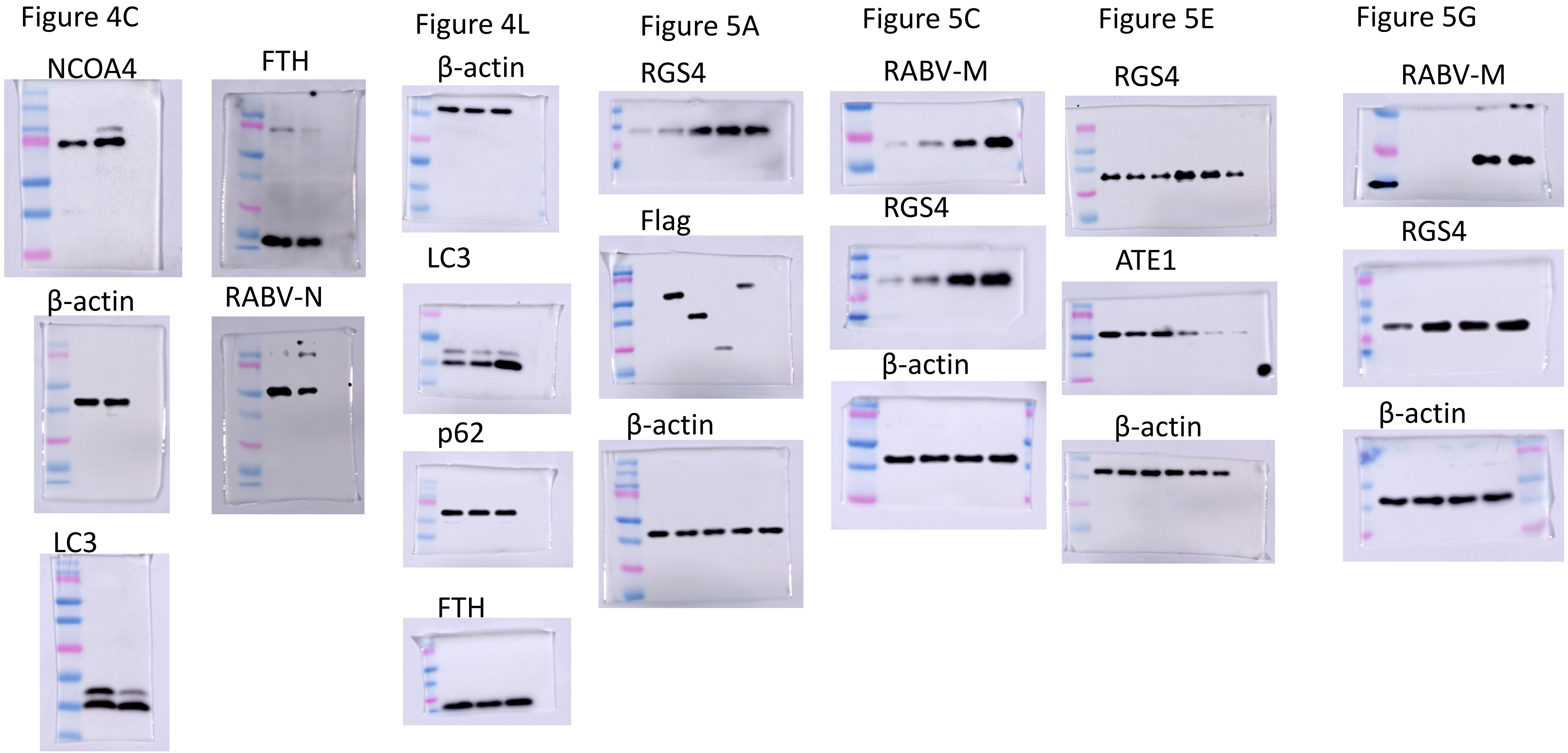
Figure 4C
NCOA4
FTH
β-actin
RABV-N
LC3
Figure 4L
β-actin
LC3
p62
FTH
Figure 5A
RGS4
Flag
β-actin
Figure 5C
RABV-M
RGS4
β-actin
Figure 5E
RGS4
ATE1
β-actin
Figure 5G
RABV-M
RGS4
β-actin

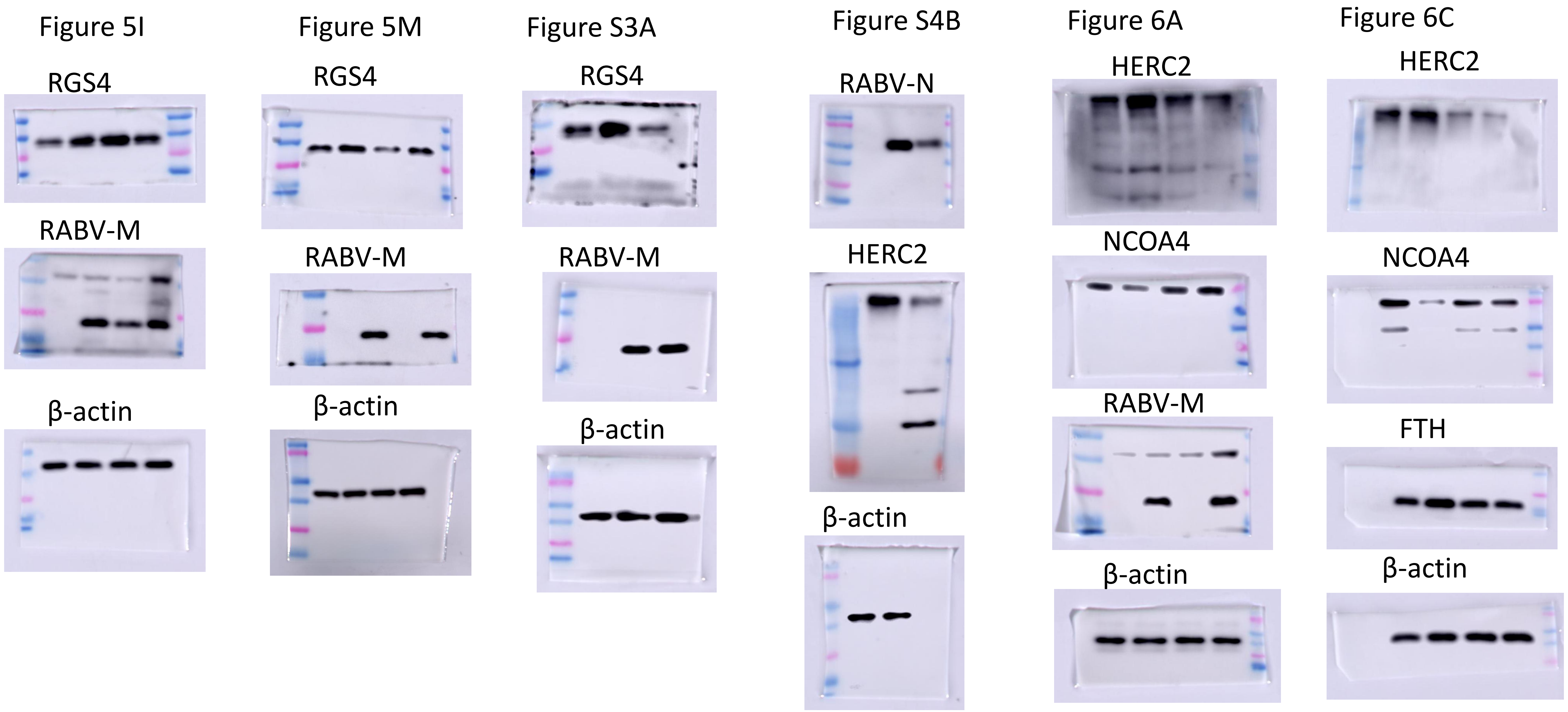
Figure 5I
RGS4
RABV-M
β-actin
Figure 5M
RGS4
RABV-M
β-actin
Figure S3A
RGS4
RABV-M
β-actin
Figure S4B
RABV-N
HERC2
β-actin
Figure 6A
HERC2
NCOA4
RABV-M
β-actin
Figure 6C
HERC2
NCOA4
FTH
β-actin

Figure 6G

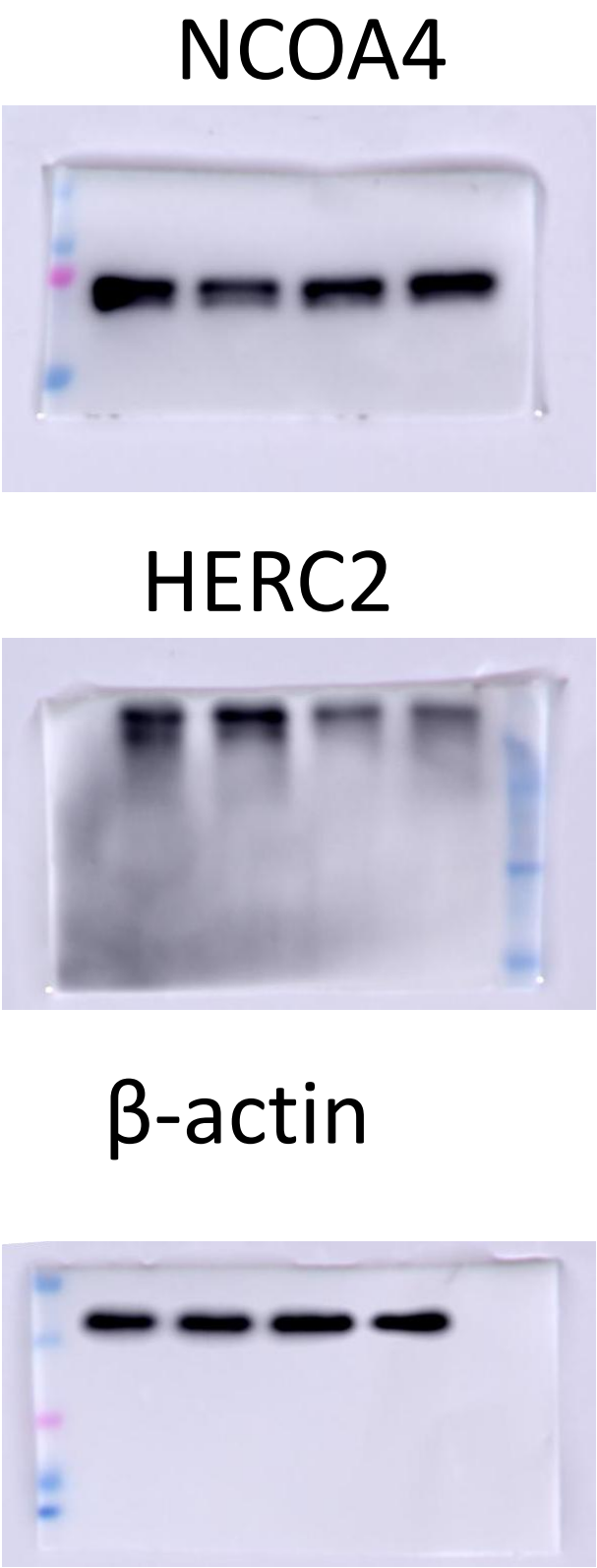

Figure 6I

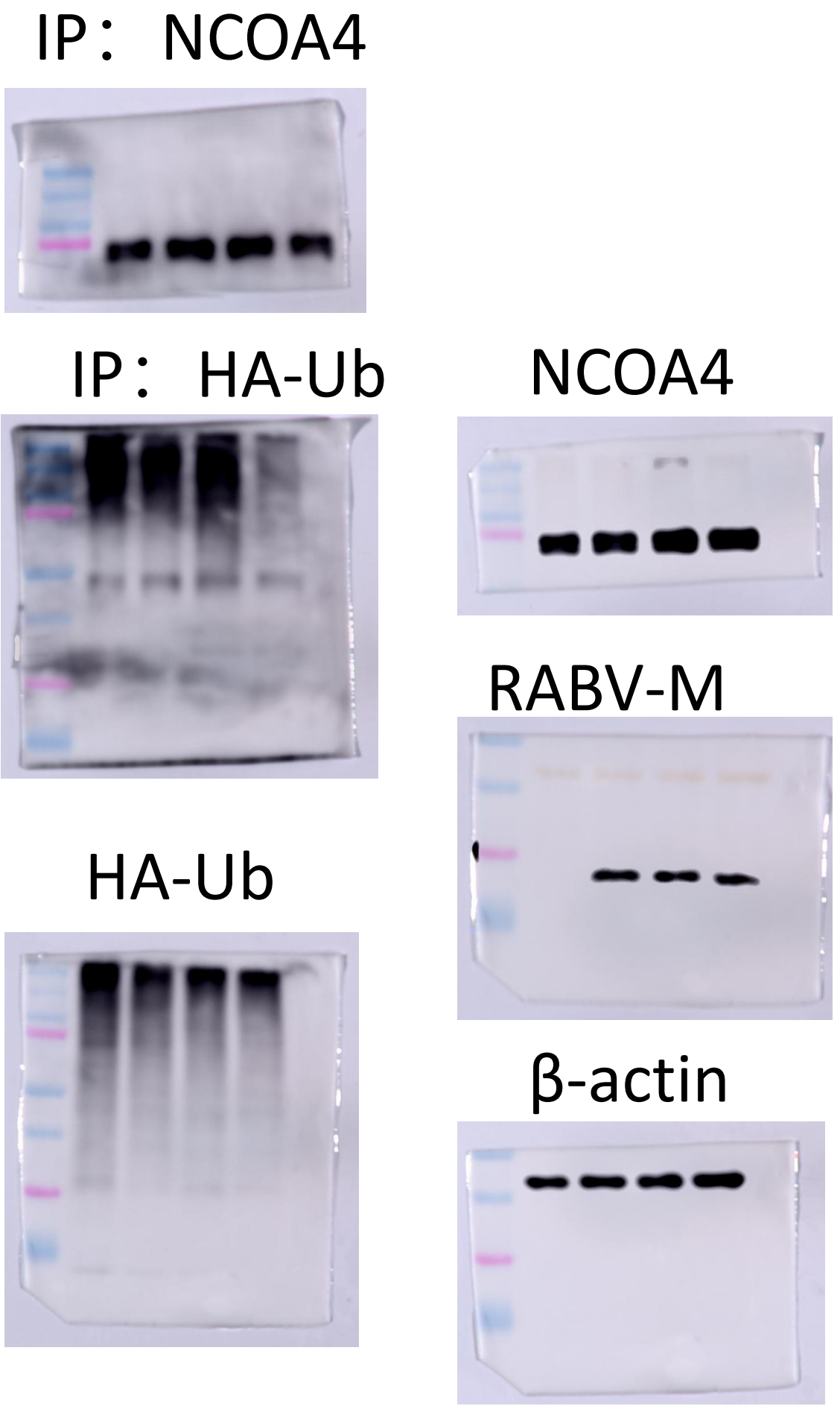

Figure 6J

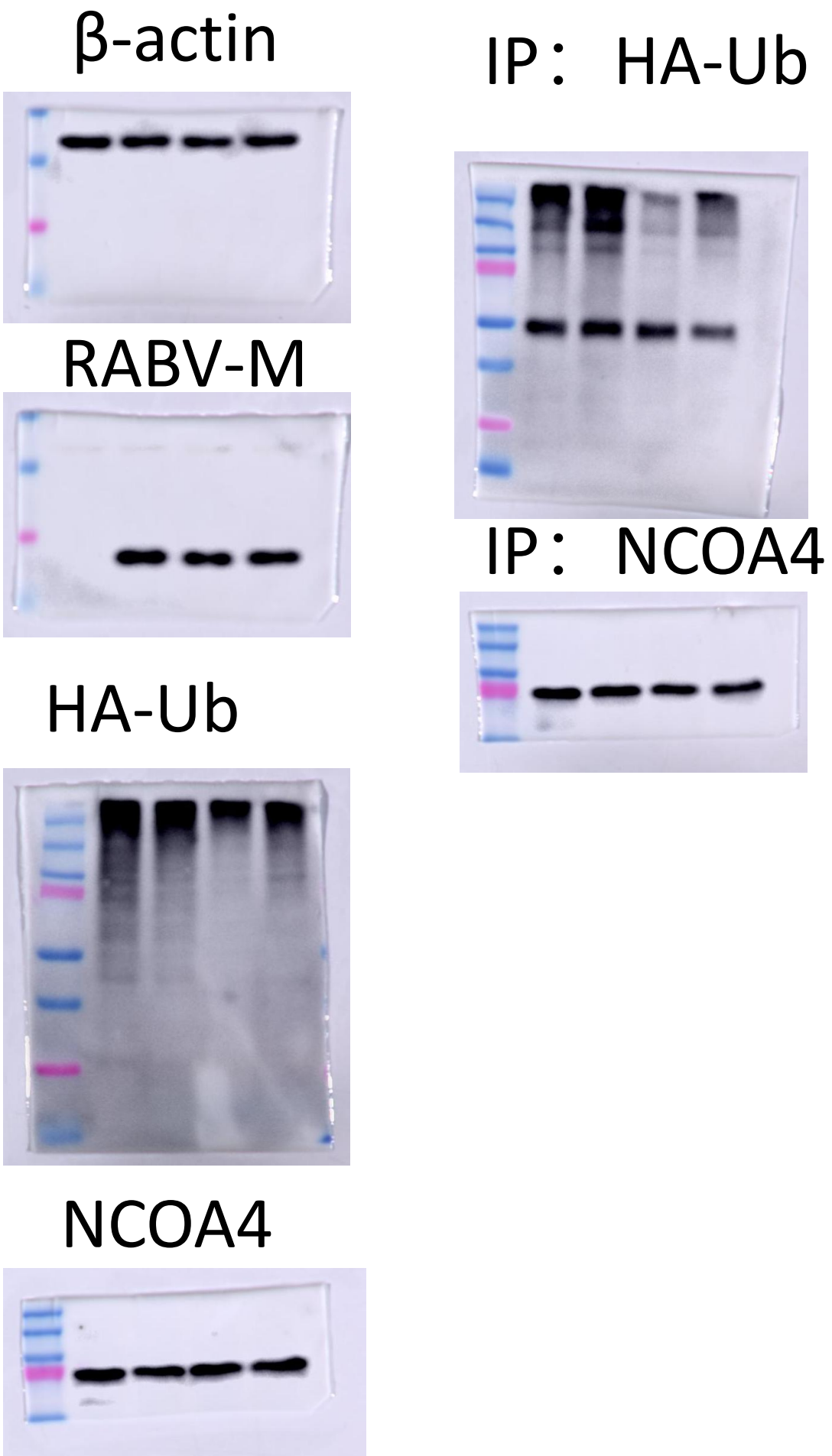

Figure 6K

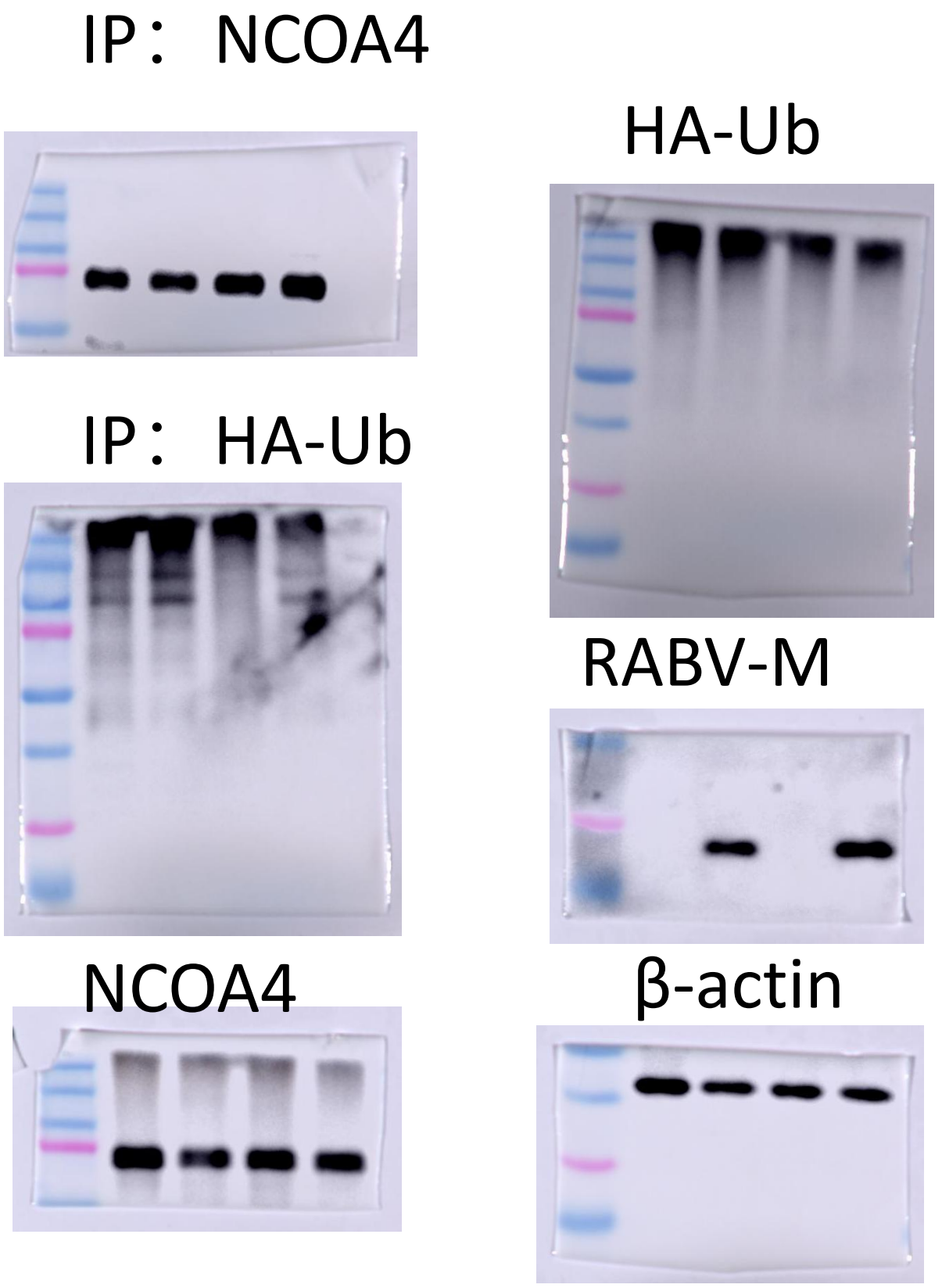

Figure 7A

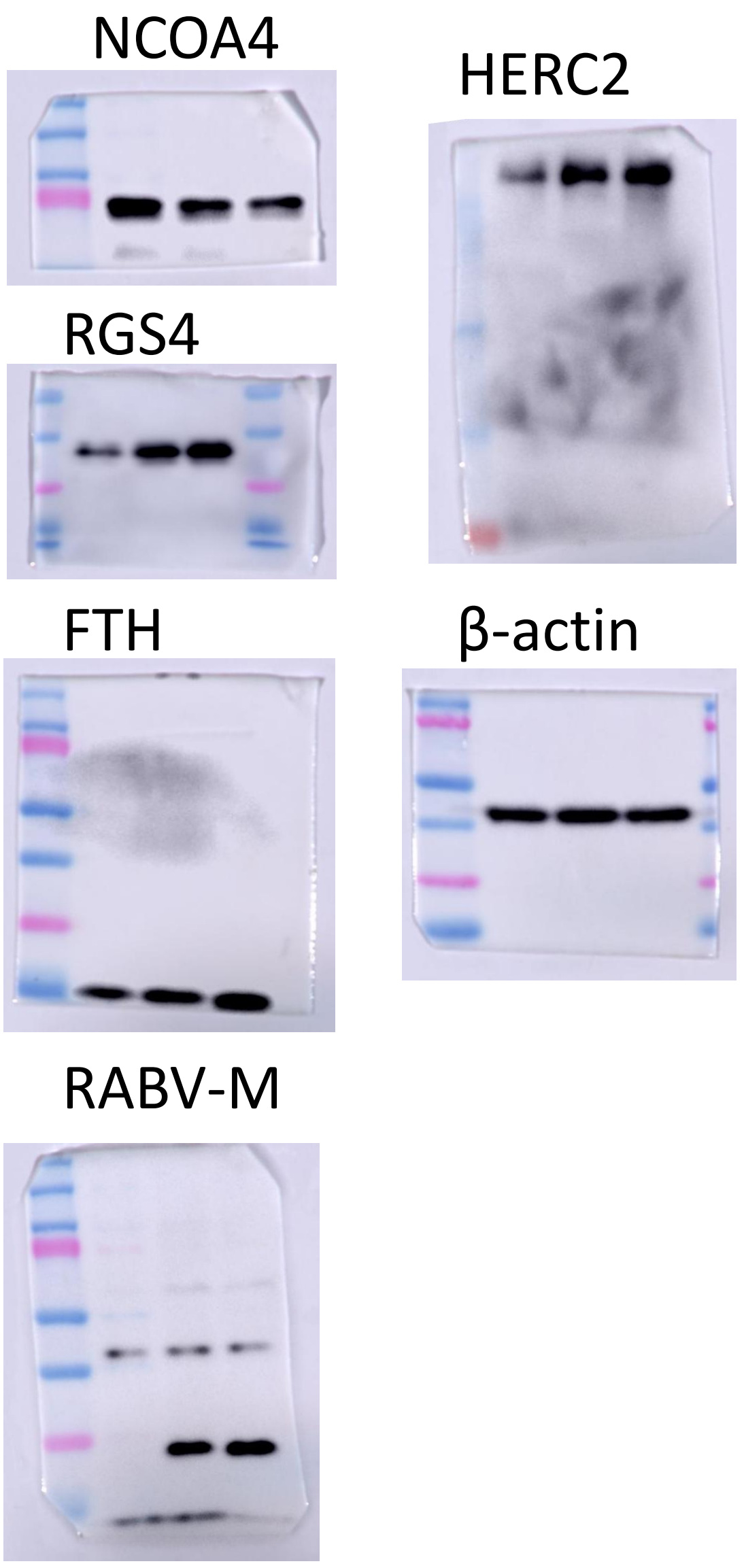

Figure 7D

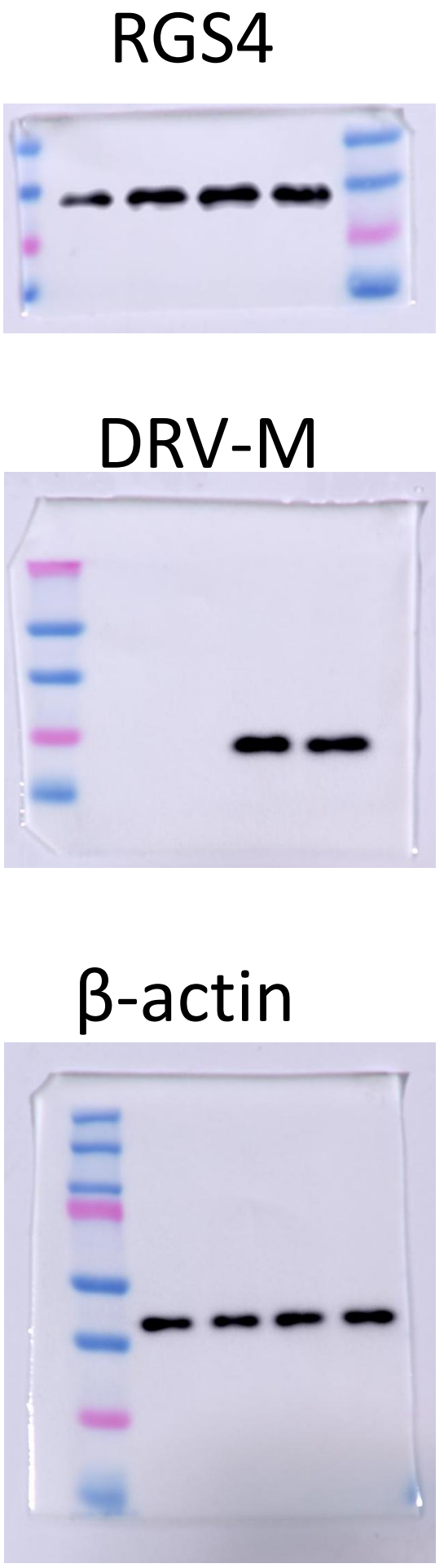

Figure 7F

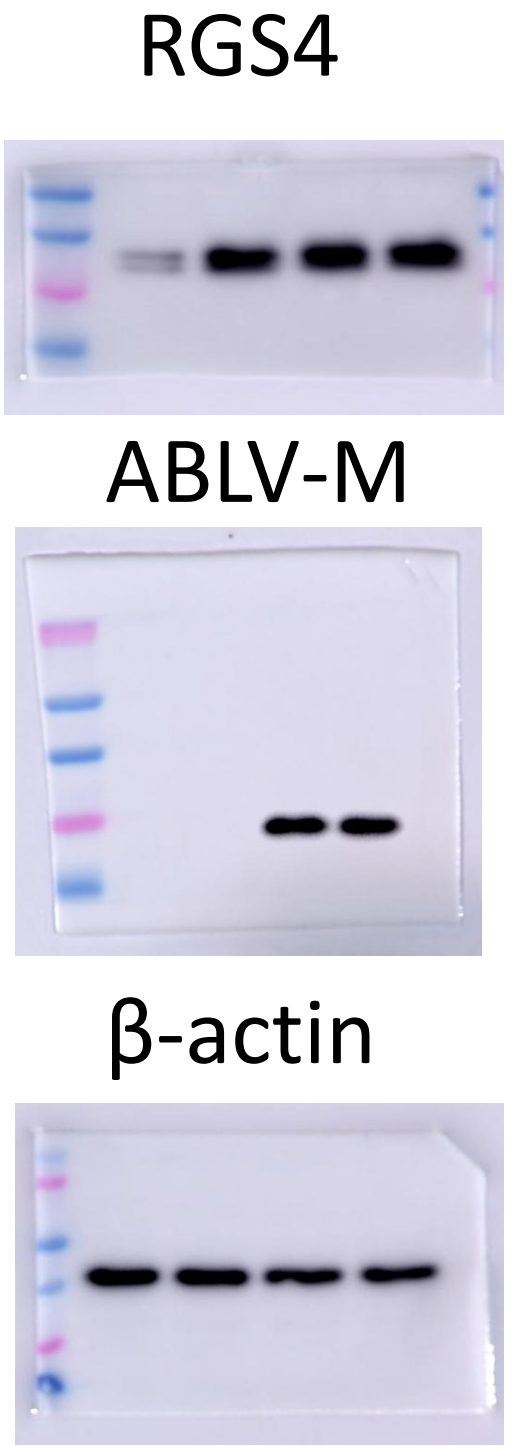

Figure 7H

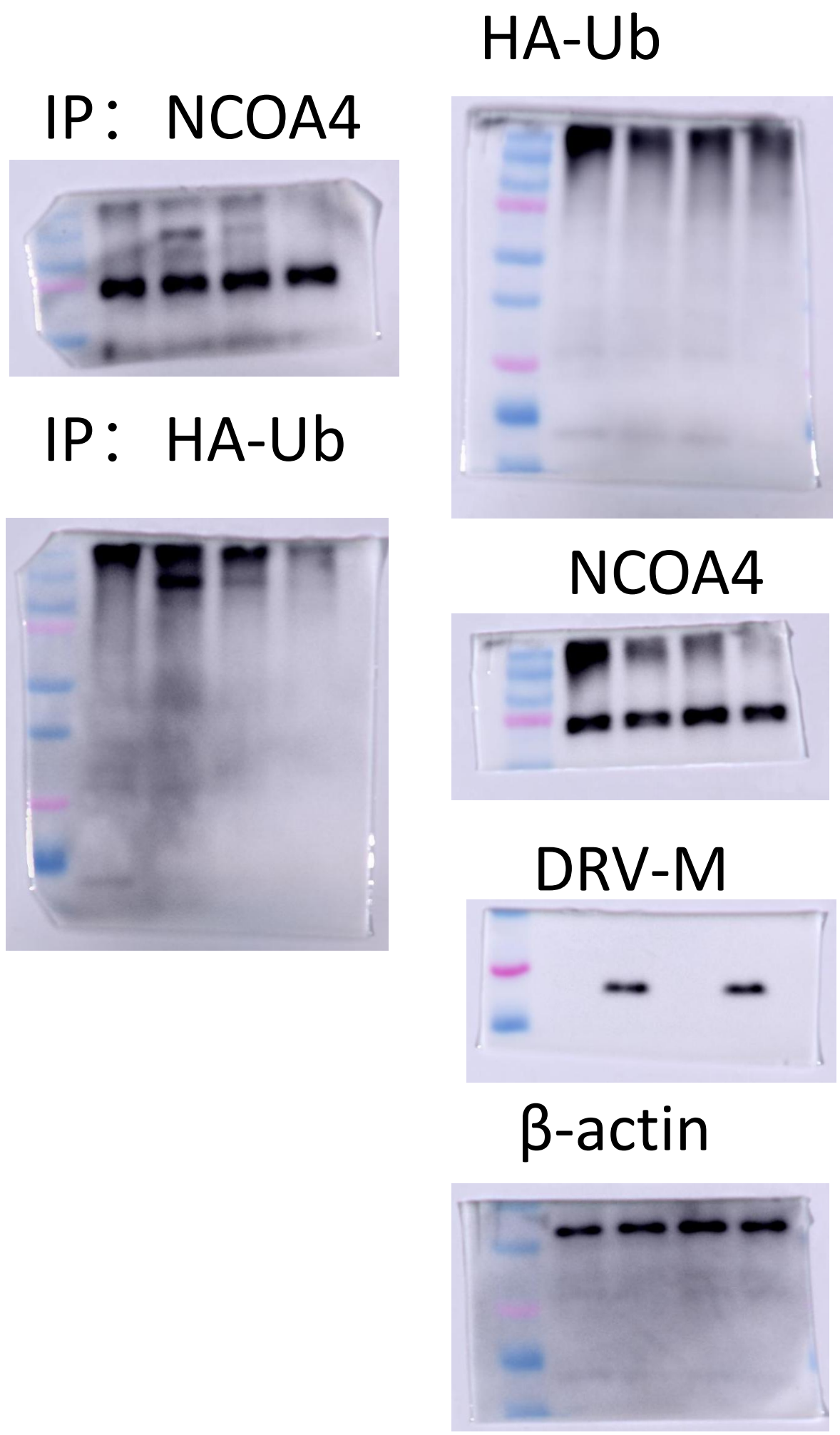

Figure 7I

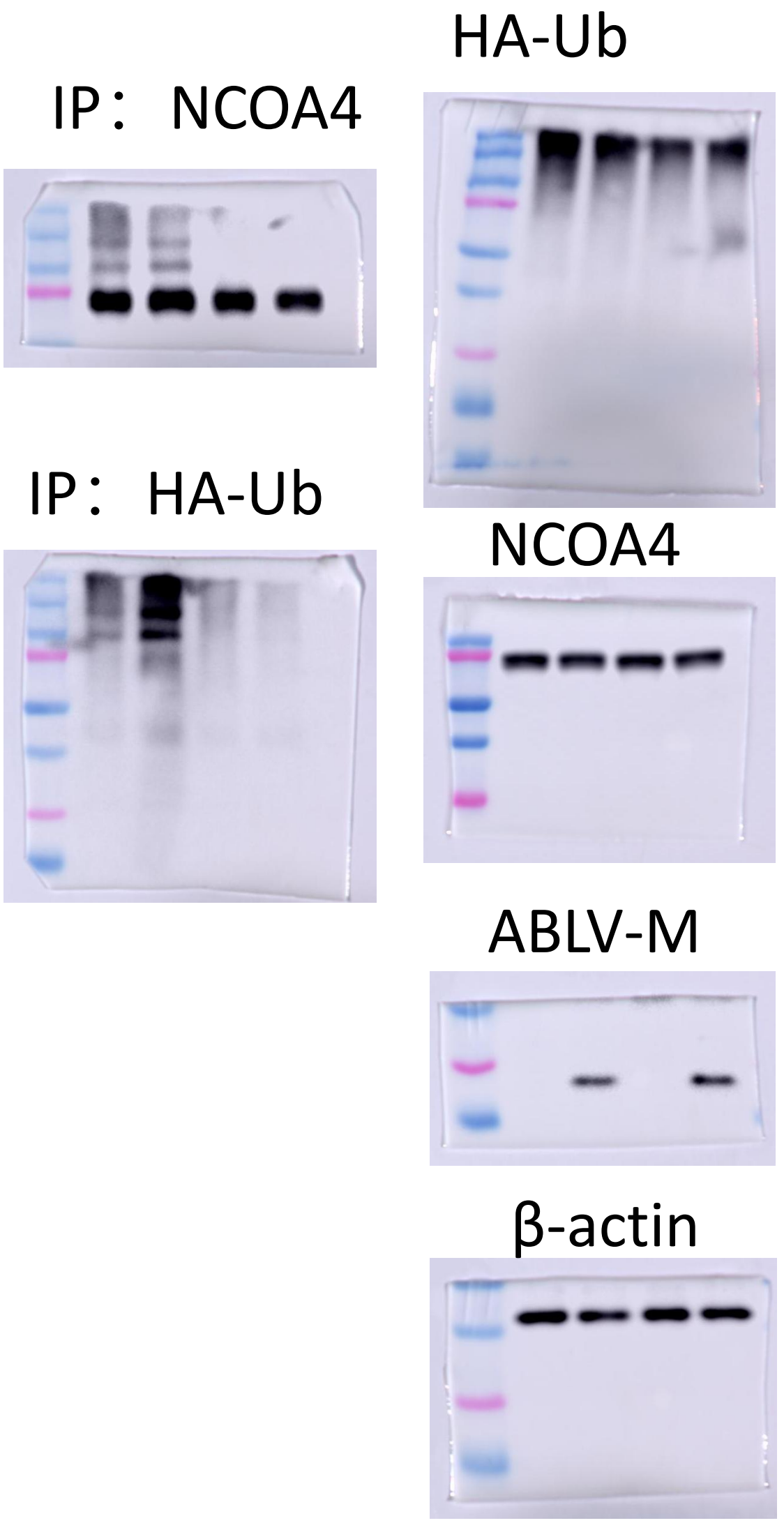

Supplement: Supplementary file 1 [file ppat.1014726.s006.pdf]
